# Discovery and characterization of novel small-molecule inhibitors targeting nicotinamide phosphoribosyltransferase

**DOI:** 10.1038/srep10043

**Published:** 2015-06-04

**Authors:** Tian-Ying Xu, Sai-Long Zhang, Guo-Qiang Dong, Xin-Zhu Liu, Xia Wang, Xiao-Qun Lv, Qi-Jun Qian, Ruo-Yu Zhang, Chun-Quan Sheng, Chao-Yu Miao

**Affiliations:** 1 Department of Pharmacology; 2 Department of Medicinal Chemistry; 3 Eastern Hepatobiliary Surgical Hospital & Institute, Second Military Medical University, Shanghai, China

## Abstract

Nicotinamide phosphoribosyltransferase (NAMPT) is a promising anticancer target. Using high throughput screening system targeting NAMPT, we obtained a potent NAMPT inhibitor MS0 (China Patent ZL201110447488.9) with excellent *in vitro* activity (IC50 = 9.87 ± 1.15nM) and anti-proliferative activity against multiple human cancer cell lines including stem-like cancer cells. Structure-activity relationship studies yielded several highly effective analogues. These inhibitors specifically bound NAMPT, rather than downstream NMNAT. We provided the first chemical case using cellular thermal shift assay to explain the difference between *in vitro* and cellular activity; MS7 showed best *in vitro* activity (IC50 = 0.93 ± 0.29 nM) but worst cellular activity due to poor target engagement in living cells. Site-directed mutagenesis studies identified important residues for NAMPT catalytic activity and inhibitor binding. The present findings contribute to deep understanding the action mode of NAMPT inhibitors and future development of NAMPT inhibitors as anticancer agents.

Nicotinamide phosphoribosyltransferase (NAMPT), also known as visfatin (a novel adipokine) or pre-B cell colony enhancing factor, plays an important role in cellular physiopathological process^1^,^2^,^3^. NAMPT converts nicotinamide into nicotinamide mononucleotide (NMN), which is subsequently converted to nicotinamide adenine dinucleotide (NAD) by nicotinamide mononucleotide adenylyltransferase (NMNAT). NAMPT is the rate-limiting enzyme in mammalian NAD biosynthesis. Much evidence supports that NAMPT has a close relationship with occurrence and development of tumor, and inhibition of NAMPT may be a novel strategy for cancer therapy^4^,^5^,^6^. Therefore, we created a high throughput screening (HTS) system targeting NAMPT^7^ based on measuring the fluorescence of nicotinamide mononucleotide (NMN) derivative resulting from the enzymatic product NMN. After a HTS study of a chemical library containing 24434 small-molecules, we obtained a potent NAMPT inhibitor MS0 that was granted with China Patent ZL201110447488.9^8^.

For NAMPT inhibitors as potential anticancer agents, several mechanisms have been proposed. Firstly, tumor cells have high NAD consumption and metabolic rate, thus they depend on NAD more than normal cells and are more sensitive to NAMPT inhibitors^6^. Secondly, NAD functions as an essential coenzyme and takes part in synthesizing many important materials of various tumors^6^. Moreover, NAD can down regulate reactive oxygen species levels to protect tumor cells^9^,^10^. NAMPT inhibitor can deplete intracellular NAD and gradually lead to cell death^5^. Besides, it has been shown that NAMPT activates endothelial angiogenesis^11^ and NAMPT inhibitor may block this process to have anticancer activity. To date, several classes of NAMPT inhibitors have been reported, and the two most advanced compounds, CHS-828 and FK866, have been progressed to clinical trials. CHS-828 is in phase I clinical trials^12^, and FK866 is in phase II clinical trials^13^,^14^. However, CHS-828 exhibits large pharmacokinetic variation, thrombocytopenia and gastrointestinal toxicity^14^. FK866 exhibits low bioavailability, rapid intravenous clearance and thrombocytopenia^13^. Thus, it is highly desirable to discover novel NAMPT inhibitors as probes or lead compounds to investigate the biological function of NAMPT and development of antitumor drug candidates.

In the current study, we identified a potent NAMPT inhibitor MS0 from our HTS platform and obtained novel structural analogues with high potency. The new inhibitors were used as chemical probes to clarify structure activity relationship, target engagement in living cells as well as the molecular action mode.

## Results

### Discovery of a potent NAMPT inhibitor MS0 by HTS

**Figure 1:**
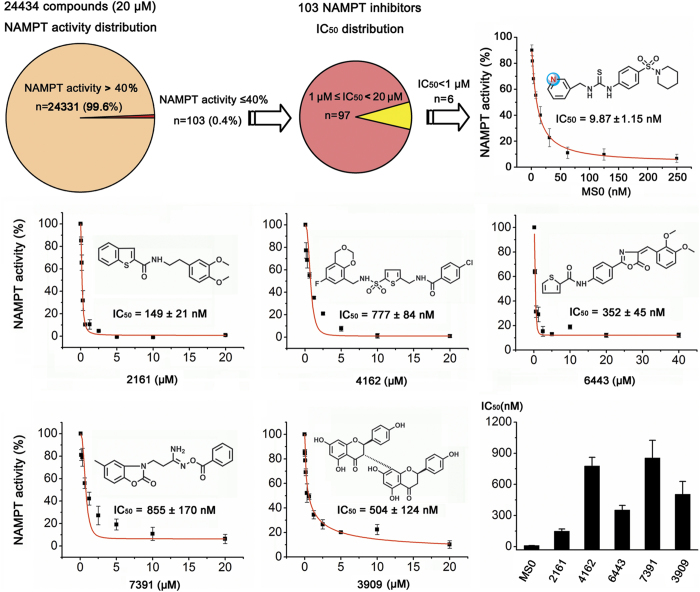
Discovery of a novel NAMPT inhibitor MS0 from the chemical library screen. Schematic illustration of discovering a novel NAMPT inhibitor MS0 by HTS in a chemical library containing 24434 small-molecule compounds. Error bars represent the s.e. of experimental triplicates.

We carried out a HTS using recombinant human NAMPT (Fig. S1) on a chemical library containing 24434 small-molecules at 20 μM. To guarantee the quality of screening, S/N ratio, CV and Z’ factors were monitored throughout the screenings, and all three indices met the requirements of HTS (Fig. S2). Most of the compounds did not significantly regulate the activity of NAMPT, and the hit rate for inhibitor (NAMPT activity ≤40%) was ~0.4% (Fig. 1). After IC_50_ determination, 6 of 103 inhibitors were validated as NAMPT inhibitors with IC_50_ less than 1 μM. Among them, MS0 (compound number 735 in the Maybridge database) was the most potent inhibitor with IC_50_ of 9.87 nM (Fig. 1, Fig. S3).

### MS0 reduces cellular NAD level and inhibits cancer cell proliferation

**Figure 2:**
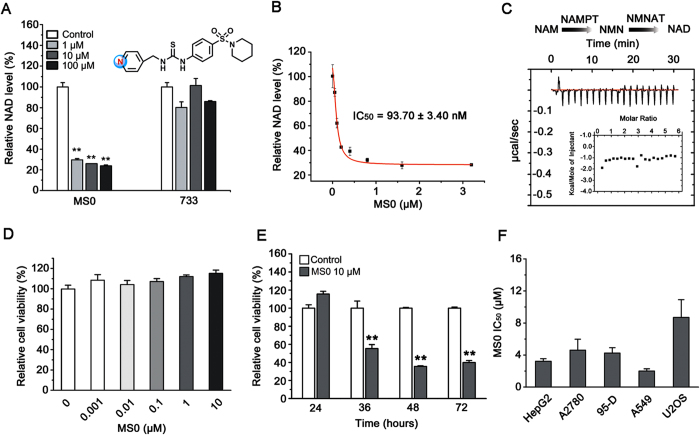
Identification of MS0 as an antitumor compound. (**A**) Effect of MS0 and its analogue 733 on NAD level of HepG2 cells at 0 ∼ 100 μM after 24 hours treatment. (**B**) Concentration response curve of MS0 on NAD level of HepG2 cells after 24 hours treatment. (**C**) ITC titration of MS0 into NMNAT does not show detectable interaction. (**D**) Effect of MS0 at 0 ~ 10 μM on HepG2 cell viability (CCK-8 assay after 24 hours treatment). (**E**) Effect of 10 μM MS0 on HepG2 cell viability (CCK-8 assay after 24, 36, 48 or 72 hours treatment). (**F**) IC_50_ of MS0 inhibition on various human cancer cell lines (SRB assay after 72 hours treatment). **P < 0.01 vs. Control. Error bars in (**A**), (**B**) and (**D**-**F**) represent the s.e. of experimental triplicates.

After incubation with human hepatocellular carcinoma cell line HepG2 for 24 hours, MS0 decreased the cellular NAD level by ~70% at 1 μM, while the structurally similar compound 733had no inhibition on NAMPT activity and did not show any effect on cellular NAD level even at 100 μM (Fig. 2A). The IC_50_ for MS0 reducing NAD level was 93.7 nM (Fig. 2B). In addition to NAMPT, NMNAT may affect the cellular NAD level (Fig. 2C). Using isothermal titration calorimetry (ITC), we did not detect an interaction between MS0 and NMNAT, thus excluding the possibility of NMNAT inhibition on NAD level by MS0 (Fig. 2C). To exclude the possibility that the decreased cellular NAD level results from the cell death, we examined the effect of MS0 on the cell viability using cell counting kit-8 (CCK-8) assay. The cell viability almost had no changes after the treatment with MS0 for 24 hours up to 10 μM (Fig. 2D), suggesting that MS0 has no direct and immediate cytotoxicity but gradually depletes the cells of some vital factor, such as NAD, that eventually triggers cell death. This viewpoint was supported by the time course of MS0 effects; MS0 treatment for ≥36 hours markedly inhibited HepG2 cell viability (Fig. 2E).

Using sulforhodamine B protein staining (SRB) assay, MS0 displayed potent growth inhibition in a dose-dependent manner in several human cancer cell lines, including hepatocellular carcinoma cell line HepG2, ovarian cancer cell line A2780, metastatic lung cancer cell line 95-D, lung adenocarcinoma cell line A549, and osteosarcoma cell line U2OS (Fig. 2F).

### Structure activity relationship studies

**Figure 3:**
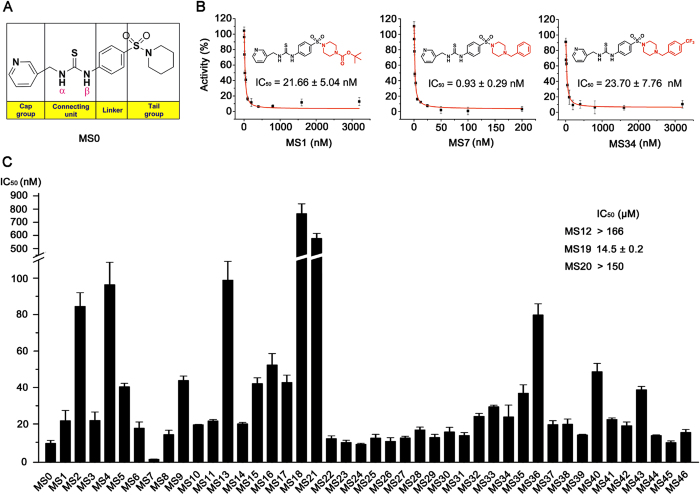
Inhibitory potency (IC_50_) for designed and synthesized MS0 analogues on NAMPT activity. MS0 and 46 novel analogues were synthesized and examined. Error bars in (**B**) and (**C**) represent the s.e. of experimental triplicates.

We investigated structure activity relationship of MS0 by designing and synthesizing 46 novel analogues and determining their IC_50_ for NAMPT inhibition (Fig. 3, Table 1 and Table S1). As stated above, moving the pyridyl nitrogen atom in the cap group from the 3-position (MS0) to the 4-position (733) led to a dramatic loss of potency in both biochemical and cell-based assays (Fig. 1, Fig. 2A), indicating the importance of the pyridyl nitrogen. Moreover, the methyl analogue MS20 without the pyridinyl group showed no NAMPT inhibition (IC_50_ > 150 μM). Similarly, compounds containing carbonyl group in the tail group (MS13, MS15, MS16 and MS20) also displayed weaker NAMPT inhibition than the corresponding sulfonamide derivatives, highlighting the importance of the sulfonamide group. In the connecting unit, modifications of this part had negative impacts on the biological activity of the resulting compounds. For example, as compared with the thiourea compound MS0, the cyanoguanidinyl derivative MS12 and guanidinyl derivative MS18 showed a dramatic decrease of the NAMPT inhibition. Similarly, the corresponding urea derivative MS14 and carbamate derivative MS17 displayed weaker potency in NAMPT inhibition. Because the pyridyl, thiourea and sulfonamide groups in inhibitor MS0 were necessary for the activity, the tail group was further optimized. The binding model of inhibitor MS0 with NAMPT revealed that there was a pocket to extend the piperdinyl group and thus could form stronger interactions. To validate the hypothesis, derivatives with phenyl, cyclopentyl amine, morpholine and 4-substituted piperazine in the tail group (compounds MS1-11) were synthesized and assayed. When the piperdinyl group of inhibitor MS0 was replaced by piperazine (MS2), phenyl (MS3), cyclopentyl amine (MS9), and morpholine (MS11), their NAMPT inhibition was decreased. Interestingly, the addition of substitutions on the piperazinyl group of compound MS2 led to the improvement of the activity again. For example, the addition of a *tert*-butoxycarbonyl group (compound MS1) resulted in about four fold increase of the NAMPT inhibition. Moreover, when the 4-position of piperazine was substituted with benzyl group, compound MS7 showed the best NAMPT inhibitory activity (IC_50_ = 0.93 nM), better than a well known NAMPT inhibitor FK866 (IC_50_ = 2.18 nM) in our HTS system. Adding substitutions on the phenyl ring of compound MS7 generally resulted in slightly decrease of the NAMPT inhibition (compounds MS22-38). In particular, compound MS36 was poorly active mainly because its butyl group was too long to be accommodated by the hydrophobic pocket. On the other hand, the heteroaromatic analogues MS39-45 were less potent than the substituted benzyl derivative MS7 because the heterocycles formed weaker hydrophobic interactions with NAMPT.

### Inhibitory effect of MS0 and its analogues on human cancer cell lines and stem-like cancer cell line

MS0 and 46 analogues were screened in three human cancer cell lines (HepG2, A549 and HCT116) for anti-proliferation effect at 10-fold concentration gradient using SRB assay. Ten compounds, namely MS0, MS1, MS23, MS28, MS31, MS32, MS33, MS34, MS35 and MS37, showed excellent cellular potency with IC_50_ below 5 μM in all three cell lines. These compounds also showed excellent *in vitro* NAMPT inhibition activity with IC_50_ mainly below 30 nM (Fig. 3C). By contrast, compounds whose NAMPT inhibition activity was above 30 nM all showed inferior cellular potency. These results indicate a good consistency between cellular and *in vitro* potency. However, there was an obvious exception that MS7 with best biochemical potency showed poor cellular potency and performed worse than MS0, MS1 and MS34 in all three cancer cell lines tested.

**Figure 4:**
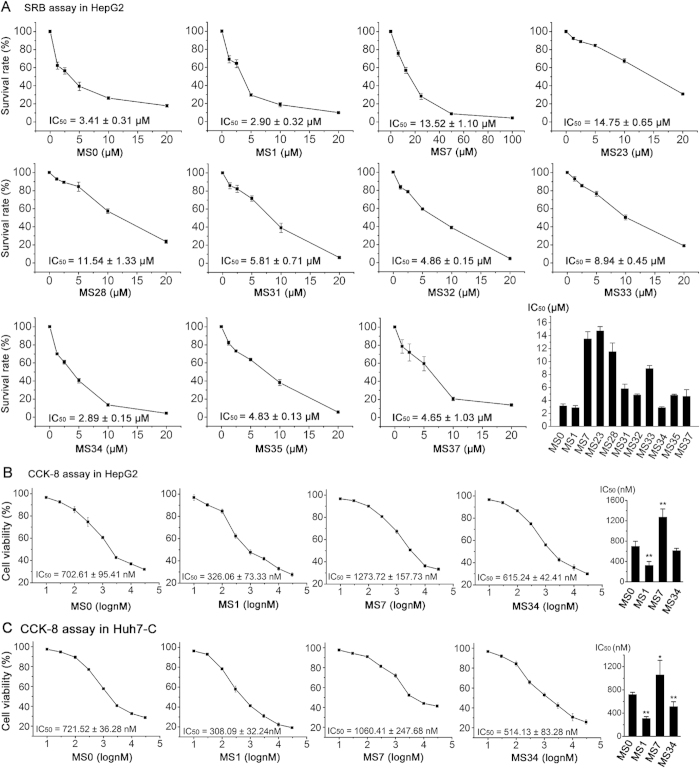
Inhibitory potency (IC_50_) for MS0 analogues on human cancer cell lines and stem-like cancer cell line. (**A**) After initial screen in three human cancer cell lines (HepG2, A549 and HCT116), 11 MS0 analogues were selected for IC_50_ determination in HepG2 cells using SRB assay after 72 hours treatment. (**B**) Further comparison was performed for the potency of 4 MS0 analogues in HepG2 cells using CCK-8 assay after 48 hours treatment. **P < 0.01 vs. MS0. (**C**) Similar effects were observed for these 4 MS0 analogues in a stem-like human hepatoma cell line Huh7-C. *P < 0.05, **P < 0.01 vs. MS0. Error bars in A-C represent the s.e. of experimental triplicates.

We then chose 11 compounds (MS7 plus 10 compounds obtained from initial screen) for second test in HepG2 cells at 2-fold concentration gradient to confirm more accurate IC_50_ values. Among them, MS0, MS1 and MS34 displayed the best growth inhibition, with IC_50_ around 3 μM, while MS7, MS23 and MS28 exhibited the worst growth inhibition with IC_50_ above 10 μM (Fig. 4A). A known NAMPT inhibitor FK866 was also examined and showed the growth inhibition with IC_50_ of 0.05 μM.

For inhibitory effects of MS0, MS1, MS7 and MS34 in HepG2 cells, CCK-8 assay also demonstrated a similar tendency (Fig. 4B) as those from SRB assay (Fig. 4A), although their IC_50_ values were much lower in CCK-8 assay than SRB assay, due to differences between the methods. Briefly, MS1 performed the best with IC_50_ of 326 nM, approximately half of the IC_50_ of MS0. MS34 has similar potency as MS0, while MS7 was the worst (1273 nM).

We next tested the potential effect of MS0, MS1, MS7 and MS34 in a stem-like human hepatoma cell line Huh7-C (Fig. S4)^15^,^16^. Results obtained in Huh7-C cells by CCK-8 assay were comparable with those in HepG2 cells, indicating relatively good sensitivity of stem-like cells to these compounds (Fig. 4C).

### Verification of NAMPT as the direct target of MS0 and its analogues in intact cell as well as cell lysate

**Figure 5:**
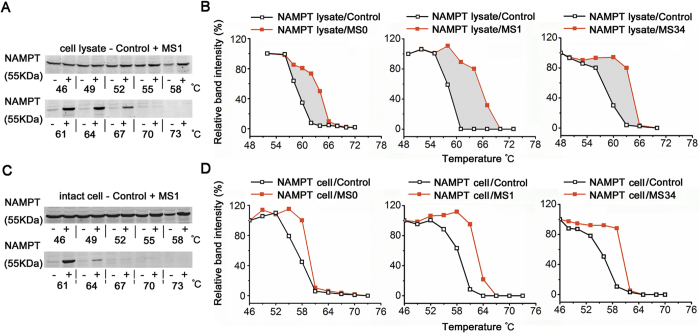
Monitoring target binding of MS0 analogues in cell lysate and in intact cell. (**A**) Representative Western blot of cellular thermal shift assay (CETSA) in cell lysate for NAMPT target with MS1 (at 100 μM). (B) CESTA melt curves in cell lysate for NAMPT target with MS0, MS1 and MS34 (all at 100 μM). (**C**) Representative Western blot of CETSA in intact cell for NAMPT target with MS1 (at 10 μM). (**D**) CESTA melt curves in intact cell for NAMPT target with MS0, MS1 and MS34 (all at 10 μM).

To verify NAMPT as the direct binding target in intact cell as well as cell lysate, we used cellular thermal shift assay (CETSA)^17^ to observe the three superior compounds MS0, MS1 and MS34 on HepG2 cells. NAMPT protein levels from cell lysate incubated with MS1 were more stable to heating as compared with the control group, indicating potent binding of MS1 with NAMPT (Fig. 5A). Similar results were also seen in MS0 and MS34. When three above compounds were added to the cell lysates, obvious right shifts in the melting curves were detected (Fig. 5B). Similar results were obtained in intact cell (Fig. 5C and D). Thus, NAMPT is confirmed as the direct binding target of the tested compounds in living cells as well as cell lysates.

### Poor target engagement in intact cell leads to the inferior performance of MS7 in hepatic cancer cells

**Figure 6:**
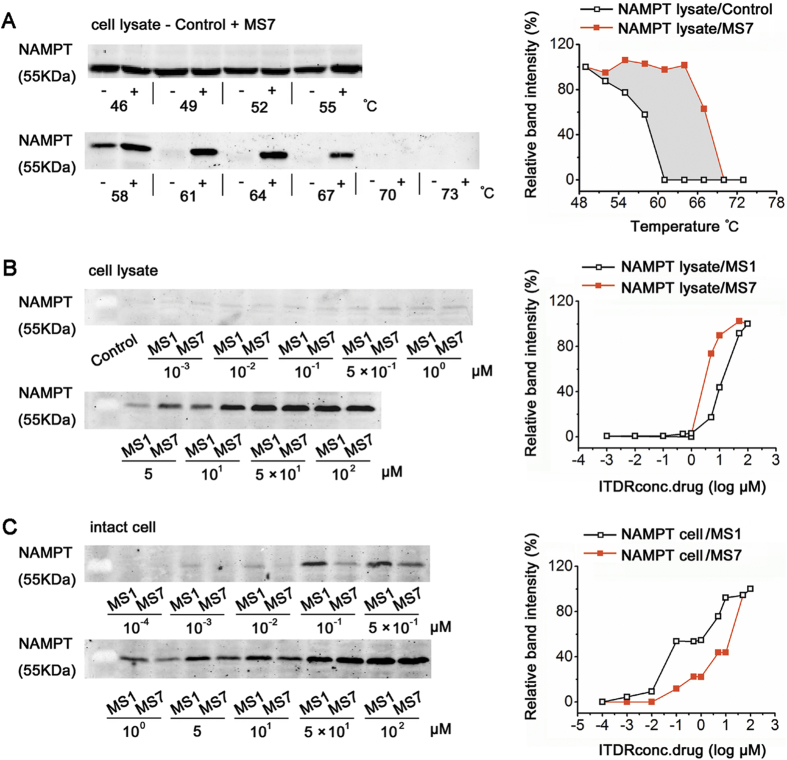
Comparison for target engagement of MS0 analogues in cell lysate and in intact cell. (**A**) CESTA melt curves in cell lysate for NAMPT target with MS7 (at 100 μM). (**B**, **C**) ITDRF_CETSA_ at 62 °C in cell lysate (**B**) or in intact cell (**C**) for NAMPT target with MS1 or MS7.

MS7 has the most potent *in vitro* NAMPT inhibition activity among all examined compounds (Fig. 3B,C). However, its inhibitory effect on tumor cells was proved repeatedly to be the worst compared with MS0, MS1 and MS34 (Fig. 4). Considering the complexity of protein regulation and target engagement in intact cell, we used CESTA to further monitor MS7 on direct target engagement in HepG2 cells. MS7 incubation induced a large thermal shift, indicating potent binding of MS7 with NAMPT in cell lysate (Fig. 6A). Moreover, when compared the shaded area of MS7 with those of MS0, MS1 and MS34 (Fig. 6A, Fig. 5B, Fig. S5), MS7 induced the largest thermal shift, which was consistent with the result that MS7 owes the most potent *in vitro* NAMPT inhibition activity. Thereafter, we used iso-thermal dose-response fingerprint (ITDRF_CETSA_) to estimate relative differences in drug concentration required to establish a similar extent of target engagement^17^, in which lysate aliquots or intact cells were exposed to different concentrations of drug with heating time and temperature kept constant. When compared with MS1, the ITDRF_CETSA_ for NAMPT in lysate treated with MS7 apparently shifted to the left, suggesting that MS7 is more potent than MS1 on NAMPT target engagement in cell lysate (Fig. 6B). On the contrary, the ITDRF_CETSA_ for NAMPT in intact cell treated with MS7 shifted to the right and yielded no less than 10-fold difference, indicating that MS7 is less potent than MS1 on NAMPT target engagement in intact cell (Fig. 6C). Moreover, the ITDRF_CETSA_ of NAMPT in cell lysate treated with MS1 gave approximately 100-fold higher value than in intact cells, suggesting a highly efficient transport of MS1 into the cell and a marked accumulation of the drug in proliferating cells. However, no significant difference was seen between ITDRF_CETSA_ of NAMPT in cell lysate and intact cell treated with MS7, indicating a relatively poor transport of MS7 into the cell.

### The molecular basis for inhibitory effect of MS0 and its analogues on NAMPT

**Figure 7:**
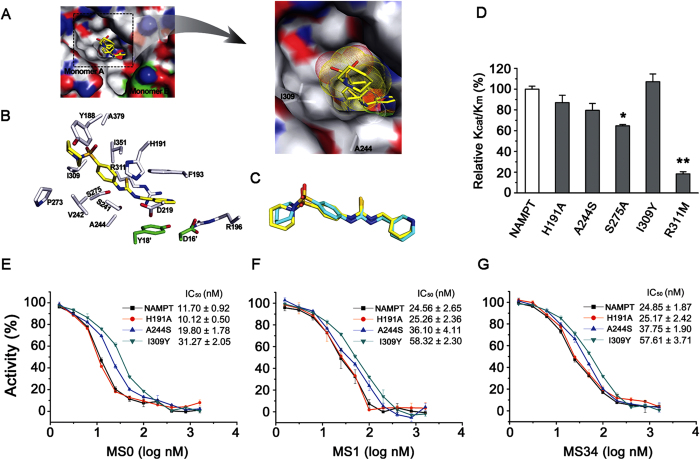
Binding mode study of MS0 analogues with NAMPT. (**A**-**B**) The potential binding mode of MS0 (yellow stick) with NAMPT. (**C**) The comparison of MS0 binding conformation in our docking model (yellow stick) with that in reported complex crystal structure (cyan stick). (**D**) Relative Kcat/Km in wild type NAMPT and 5 mutants. *P < 0.05, **P < 0.01 vs. NAMPT. (**E**-**G**) IC_50_ of MS0 (**E**), MS1 (**F**) and MS34 (**G**) on wild type NAMPT and 3 mutants (H191A, A244S and I309Y). Error bars in (**D**-**G**) represent the s.e. of experimental triplicates.

To provide insight into the molecular basis of MS0 inhibiting NAMPT, we performed molecular modeling study based on the crystal structure of NAMPT in complex with FK866 (PDBID: 2GVJ)^18^ and proposed the most reasonable binding mode on the basis of SAR (Fig. 7A,B). After the completion of this work, Zheng *et al.* reported a crystal structure (PDBID: 4JR5)^19^ of NAMPT in complex with MS0, which reveals nearly the same binding mode as our model (Fig. 7C). Both of them consistently identified key residues interacting with MS0, mainly including Y18, A244, S241, S275, I309, Y188, I351, H191, F193 and R311 (Fig. 7B). To validate the importance of these residues for the binding, we mutated several residues around the bound MS0, such as H191A, A244S, S275A, I309Y and R311M. The enzyme kinetics study (Fig. 7D, Fig. S6) revealed that S275A or R311M mutation significantly impaired the enzyme activity, indicating both S275 and R311 are important for the catalytic activity of NAMPT. While H191A, A244S or I309Y mutation showed almost the same kinetic parameters compared to those of wild-type NAMPT and did not alter the enzyme activity. Therefore, we evaluated the inhibitory effect of MS0 against H191A, A244S and I309Y mutant under the exactly same condition we used for the wild-type NAMPT. Comparing with the IC_50_ value against wild-type NAMPT (11.70 ± 0.92 nM), MS0 showed about 2-fold increase in IC_50_ (19.80 ± 1.78 nM) for A244S mutant and 3-fold (31.27 ± 2.05 nM) for I309Y mutant, but no change (10.12 ± 0.50 nM) for H191A mutant (Fig. 7E), indicating that the substitution by bulkier residue at A244 and I309 may shrink the tunnel, perturb the binding of MS0 and thereby lower the inhibition potency of MS0 against the mutants, while substitution by an Alanine at H191 nearly does not interfere with binding of MS0 at NAMPT. We also examined the inhibitory effect of two analogues, MS1 and MS34, on the three mutants (Fig. 7F,G), both inhibitors showed similar inhibition profile against mutants versus wild-type: about 2 to 3 fold decrease for A244S and I309Y but no change for H191A.

## Discussion

Based on our previously established HTS targeting NAMPT, we obtained a NAMPT inhibitor MS0 with high potency. Our HTS was developed based on a fluorometric method for NAMPT activity assay by measuring the fluorescence of nicotinamide mononucleotide (NMN) derivative resulting from the NAMPT enzymatic product NMN through simple chemical reactions^7^. This method is sensitive, simple, quick and cost-effective. In contrast to our HTS method applied here, most reported NAMPT inhibitors including FK866 were discovered indirectly through cellular phenotypic screening and then molecular target identification^5^,^20^,^21^. Using our experimental assay directly targeting NAMPT, we identified a highly potent NAMPT inhibitor MS0 and demonstrated its activity against multiple human cancer cell lines, which was granted with China Patent ZL201110447488.9^8^. Interestingly, Zheng *et al.* independently reported the same compound with similar biochemical potency (IC_50_ = 7 nM) by in silico virtual screening and demonstrated its activity against an ovarian cancer cell line A2780^19^,^22^. In our study, novel structural analogues were synthesized and used as chemical probes to clarify structure activity relationship; SRB, CCK-8 and NAD assays were used to investigate the inhibitory potency and mechanism of MS0 and its analogues as antitumor compounds; ITC and CESTA were used to verify the target specificity and target engagement in living cells; and finally, combined mutagenesis and enzyme kinetics studies were used to elucidate the molecular action mode.

In addition to the fact that MS0 was proved to be highly effective against multiple human cancer cell lines, many MS0 analogues were also validated to have comparable antitumor performance. Surprisingly, MS0 and several of its analogues were further proved to have high potency against a stem-like human hepatoma cell line. So far, there are no data available on cancer stem cells regarding NAMPT inhibitors. Increasing evidence supports that cancer stem cells have close relationship with cancer development, metastasis and recurrence. Cancer stem cells are insensitive to anticancer drugs^23^. Therefore, our findings indicate that MS0 and some analogues might have potential efficacy against cancer stem cells which is worth further studying.

Chemical compounds verified to bind with desired targets *in vitro* or monitored indirectly by studying downstream cellular responses do not necessarily bind with the same target in intact cell or *in vivo*. Chemical compounds may interact with multiple targets simultaneously, or they could be off-targets. In our previous study^7^, we reported a NMNAT inhibitor gallotannin^24^ also had good NAMPT inhibition activity (IC_50_ = 1.3** **±** **0.1 μM). Therefore, NAMPT inhibitors identified from HTS have the possibility to act at NMNAT, which also results in NAD down regulation. As a case in point, a proposed PARP-1 inhibitor iniparib reached phase III clinical trials, where it showed no efficacy, and was subsequently shown to lack activity against PARP-1 in living cells. Using CESTA method further eliminated the physical binding of iniparib with PARP-1^17^. Thus, it is important to detect the direct binding of compounds with targets in intact cell and to eliminate possibility for activities at off-targets. In the current study, we further investigate the three most active compounds MS0, MS1 and MS34 and proved that they directly bound with NAMPT in intact cell using CESTA. We also excluded the other possible target NMNAT through ITC.

In our study, most cell results agreed with *in vitro* biochemical results. However, there exists an obvious exception that although MS7 had best biochemical potency and performed well in cell lysate experiments, it was proved to have poor cellular activity. Previously there were many similar examples regarding the difference between *in vitro* and *in vivo* experiments, but they were hard to explain. A recently developed method called CETSA^17^ may give a resolution for this kind of problem. CETSA was based on the biophysical principle of ligand-induced thermal stabilization of target proteins. Taking advantage of this method, it is feasible to directly monitor target engagement in living cells as well as cell lysates. Furthermore, it is also possible to estimate relative differences in drug concentration required to establish a similar extent of target engagement. This is very important, because the target engagement extent of drug in living cells is theoretically consistent with drug biological activity *in vivo*. Based on this method, we proved that MS7 showed better target engagement in cell lysate but worse target engagement in living cells than MS1, providing a reasonable explanation for the poor cellular activity of MS7. Thus, for the first time, our study provides an example using CESTA to explain the difference between *in vitro* and cellular experiments.

We used mutagenesis and kinetics study to investigate the molecular basis for inhibitory effect of MS0 and its analogues on NAMPT. Using five single-point mutants of NAMPT, we validated two residues at the entrance of (I309) and within (A244) the tunnel towards active site involved in the interaction of the compound with NAMPT, and substituting either one with bulkier residue (I309Y or A244S) reduced the binding affinity of the examined compounds. We noted that substitution by an Alanine at H191 (H191A) did not affect the inhibitory effect of MS0 or its analogues on NAMPT in our study. However, substitution by an Arginine at H191 (H191R) was reported to reduce inhibitory effect of a known NAMPT inhibitor FK866 on NAMPT and cause *in vitro* and *in vivo* resistance^25^. Thus, further study using H191R might reveal the potential clinical significance of MS0 and its analogues upon cancer cell resistance.

We also found two residues (S275 and R311) important for NAMPT catalytic activity, and their single-point mutant (S275A or R311M) had significantly low activity. These results provide novel insights for NAMPT basic research, and may help to understand potential NAMPT mutant dysfunction in certain pathological state.

In summary, we discovered a potent NAMPT inhibitor MS0 by HTS directly targeting NAMPT, and elucidated its structure activity relationship and molecular binding mode. MS0 and several novel analogues were demonstrated to have potent anticancer activity on multiple human cancer cells and stem-like cancer cells. These small-molecules specifically bound NAMPT target in living cells. We provided the first chemical example MS7 explaining the difference between *in vitro* and cellular activity by comparing its target engagement in cell lysate with in intact cell. Site-directed mutagenesis studies identified important residues for NAMPT catalytic activity and inhibitor binding. These findings of the present study contribute to the steady accumulation of knowledge on NAMPT as well as to the future development of NAMPT inhibitors as anticancer agents.

## Methods

### Chemicals

A chemical library used for high throughput screening contains 24434 small-molecules, including 9234 from the National Compound Resource Center (Shanghai, China), 14400 from Maybridge and 800 from an in-house compound collection. Compounds MS0 and 46 novel analogues were synthesized by our research group detailed in Supplemental Materials and Methods.

### Plasmids construction and protein expression

cDNA sequence of human NAMPT was amplified by PCR from pGex-6p-3-hNAMPT plasmid (kindly gift from Dr. Shui-Qing Ye in University of Missouri). The PCR products were digested and cloned into pET21a+ vector using NdeI and XhoI restriction enzyme. Point mutation was introduced by quick change site-directed mutagenesis method using the constructed pET21a+-hNAMPT plasmid as a template. Primer pairs were listed in Supplemental Materials and Methods. All the mutations were validated by DNA sequencing. His-tagged NAMPT wild-type, NAMPT mutants and NMNAT1 were expressed and purified by our previous methods^7^,^26^ detailed in Supplemental Materials and Methods and Fig. S1.

### High throughput screening (HTS)

HTS was performed using our previously reported method^7^. 0.5 μl stock of each compound (1 mM DMSO stock) was transferred to a 96-well PCR plate for screening. In the primary screening, 5 ng NAMPT in 20 μl reaction buffer [0.4 mM phosphoribosylpyrophosphate (PRPP, Sigma), 2 mM ATP, 0.02% BSA, 2 mM DTT, 12 mM MgCl_2_ and 50 mM Tris-HCl (pH = 7.5)] was added into each well, the plate was incubated at 37 °C for 5 min, then 4.5 μl substrate of NAM was added to initiate the enzyme reaction, resulting in a final concentration of 2% DMSO, 2 μg/ml NAMPT, 0.2 μM NAM and 20 μM compound. After reacting at 37 °C for 15 min, the enzyme reaction was terminated by heating at 95 °C for 1 min and cooling in an ice bath. The product of NMN was detected through the following approach: after adding 10 μl 20% acetophenone in DMSO and 10 μl 2 M KOH into each well, the mixture was vortex-mixed and kept in ice bath for 2 min. Then 45 μl 88% formic acid was added and the mixture was incubated at 37 °C for 10 min. Finally, 85 μl mixtures in each well were transferred into a flat-bottom 96-well black plate (Greiner), and the fluorescence (F) was measured using a Tecan Infinity M200 plate reader (Tecan Group Ltd.) by setting the excitation and emission wavelength to 382 nm and 445 nm respectively. The last row of each 96-well plate includes six wells of background controls and six wells of reference controls. The relative enzyme activity (Activity%) regulated by specific compound was calculated according to equation (1):
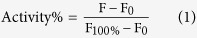
F_0_ was the averaged fluorescence of six background controls, representing zero activity from a simulated enzyme reaction with only NAMPT but no NAM and compound; F_100%_ was the averaged fluorescence of six reference controls, representing 100% activity from intact enzyme reaction without compound perturbation.

Compounds with Activity% less than 40% were considered as inhibitors and subjected to a secondary screen inhibition validation, in which another background control (F_C0_), a simulated enzyme reaction with NAMPT and compound but no NAM, was introduced to eliminate the direct and/or indirect influence from compound. At this stage, the Activity% was calculated according to equation (2):
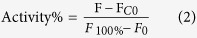


In the screening, the signal-to-noise (S/N) ratio was calculated using the equation: (Mean _signal _- Mean _background_)/SD _background_^27^. Coefficients of variation (CV) were the ratio of SD to mean. The Z’ factor was determined by equation (3)^28^:
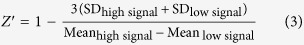


### Determination of IC50 for NAMPT inhibitors

To determine the IC_50_ of inhibitors, 5 μl compound solutions (containing 10% DMSO) with various concentrations were added into 96-well plate. The plate was incubated at 37 °C for 5 min after addition of 16.5 μl reaction buffer containing NAMPT. The enzyme reactions were initiated by 4.5 μl NAM (1.11 μM) following NMN measurement as described above. The IC_50_ values were determined by non-linear fitting of the concentration-dependent curves with the four-parameter IC_50_ logistic equation.

### Isothermal titration calorimetry (ITC)

Thermodynamic parameters of small molecule binding to protein were determined using a MicroCal VP-ITC calorimeter^29^. NMNAT protein solutions were ultrafiltrated in Amicon Ultra-0.5 Centrifugal Filter Unit (Merck Millipore) against buffer [20 mM Tris (pH 7.5), 20 mM NaCl], which was subsequently used to prepare a matched compound solution. ITC data collected for MS0 were acquired in 5% DMSO to improve compound solubility. Each isotherm was recorded by injecting 554 μM MS0 into 25 μM solutions of protein. Measurements were performed at 25 °C with spacing of 90 s between injections. Background signal (calculated as a mean value) generated by addition of MS0 to buffer was subtracted prior to analysis on Origin 7.5 using software supplied by the manufacturer.

### Cellular thermal shift assay (CETSA)

CESTA was performed as previously described^17^ and detailed in Supplemental Materials and Methods. Briefly, in the cell lysate CETSA experiments, the cell lysates were collected, diluted and divided into two aliquots, treated with drug or diluent as control. After incubation for 30 min at room temperature the respective lysates were divided into smaller aliquots and heated individually at different temperatures (Veriti thermal cycler, Applied Biosystems/Life Technologies). The heated lysates were centrifuged and the supernatants were analyzed by sodium dodecyl sulfate polyacrylamide gel electrophoresis (SDS-PAGE) followed by Western blot analysis.

In the intact cell experiments, treated cells were exposed to a drug for 3 h and were harvested. Equal amounts of cell pellets were heated as previously described. The soluble fractions were isolated and analyzed by Western blot analysis as described above.

### Binding mode study of MS0 with NAMPT

AutoDock_Vina1.1.2 program was used to build the binding mode of MS0 with NAMPT. The 3D-structure of MS0 was modeled and energy-minimized in Chem3D program, and the coordinates of NAMPT (PDBID: 2GVJ) were retrieved from the Protein Data Bank website. Both structures of MS0 and NAMPT were pre-processed in AutoDockTools1.5.4^30^, such as merge non-polar hydrogens, add Gasteiger charges, set rotatable bond for MS0, add solvation parameter, and so on. The docking space of 15 × 15 × 30 Å^3^ was visually set around the binding site of FK866, the parameter of exhaustiveness, num modes and energy range was set to 20, 1000 and 5 respectively, and the default values were used for the other parameters.

### Statistical analysis

Data are expressed as the mean ± s.e. Statistical comparisons between two groups were performed by Student’s t test. Comparisons among several groups (≥3 groups) were performed by analysis of variance followed by Tukey’s post hoc test. Statistical significance was set at P < 0.05.

For a description of other materials and methods used in this study, see the Supplemental Materials and Methods.

## Additional Information

**How to cite this article**: Xu, T.-Y. *et al*. Discovery and characterization of novel small-molecule inhibitors targeting nicotinamide phosphoribosyltransferase. *Sci. Rep.* **5**, 10043; doi: 10.1038/srep10043 (2015).

## Supplementary Material

Supplementary Information

## Figures and Tables

**Table 1:**
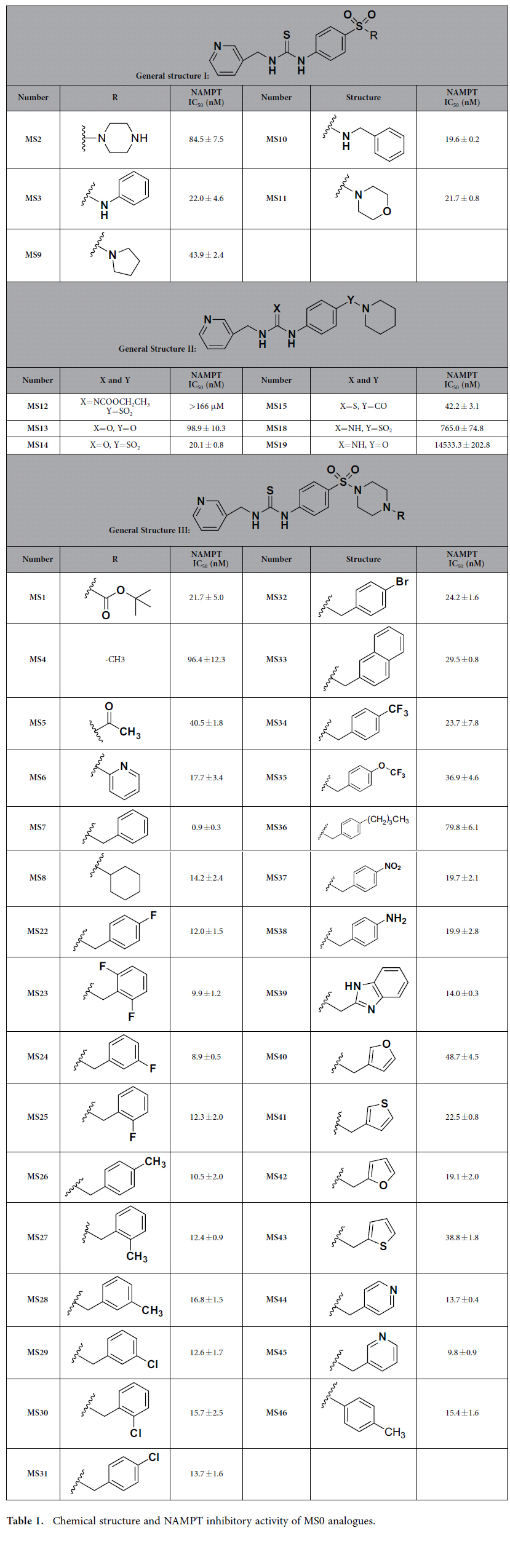
Chemical structure and NAMPT inhibitory activity of MS0 analogues.
