# Supplementary material for: Discovery and characterization of novel small-molecule inhibitors targeting nicotinamide phosphoribosyltransferase
Source: Sci Rep. 2015 Jun 4;5:10043. doi: 10.1038/srep10043 (PMC4603696; doi:10.1038/srep10043)
Supplement: Supplementary Information [file srep10043-s1.pdf]

Supplemental Information

Discovery and characterization of novel small-molecule inhibitors targeting nicotinamide phosphoribosyltransferase

Tian-Ying Xu[1], Sai-Long Zhang[1], Guo-Qiang Dong[2], Xin-Zhu Liu[1], Xia Wang[1], Xiao-Qun Lv[1], Qi-Jun Qian[3], Ruo-Yu Zhang[1]*, Chun-Quan Sheng[2]* & Chao-Yu Miao[1]*

[1]Department of Pharmacology, [2]Department of Medicinal Chemistry and [3]Eastern Hepatobiliary Surgical Hospital & Institute, Second Military Medical University, Shanghai, China.

The first three authors contributed equally to this work.

*Corresponding author:

E-mail: cymiao@smmu.edu.cn, shengcq@hotmail.com or violeter_80@163.com

This file includes:

1. Supplemental Data (6 Figures and 1 Table, Page 1-Page 14)

2. Supplemental Materials and Methods (Page 15-Page 35)

3. Supplemental References (Page 36-Page 37)

Supplemental Data

Fig. S1. SDS-PAGE and Coomassie Blue staining (A) and Western blot analysis (B, C) of the purified human NAMPT protein. The amount of purified human NAMPT protein (green) loaded for western blot analysis is 18 μg in (B) and 600 ng in (C).

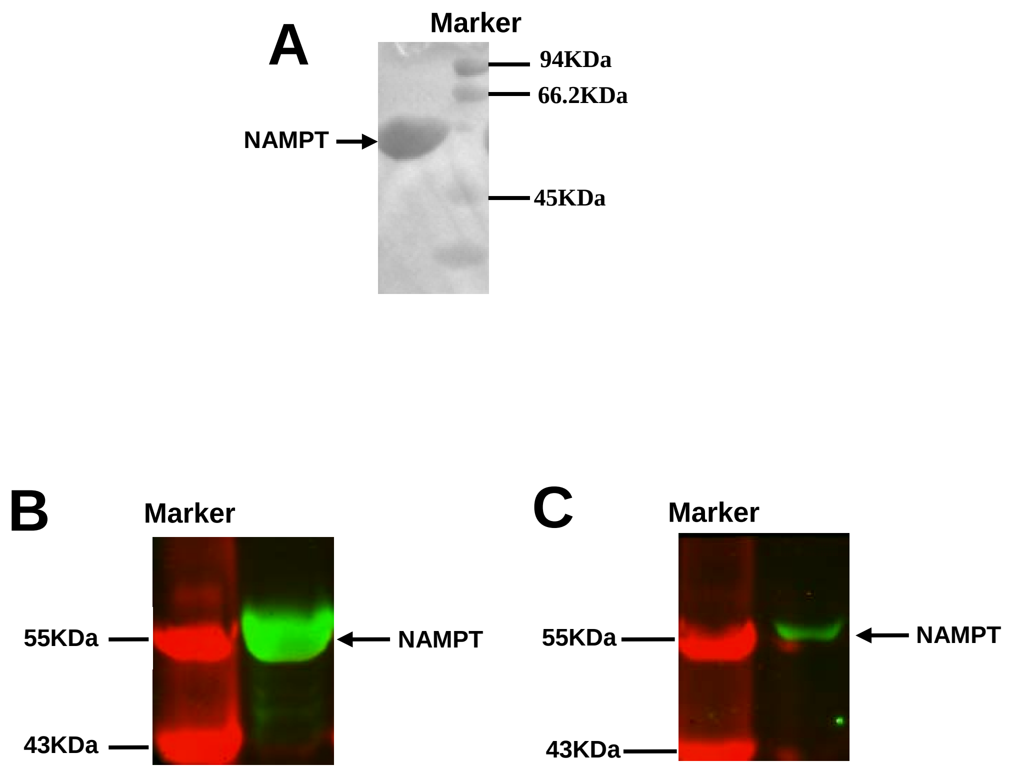

Fig. S2. Parameters in high throughput screening. The signal-to-noise (S/N) ratio was calculated using the equation: (Mean $_{signal}$ -Mean $_{background}$) / SD $_{background}$. Each coefficient of variation (CV) was the ratio of the SD to the mean. The Z’ factor was determined by equation:

$$Z' = 1 - \frac{3(SD_{\text{high signal}} + SD_{\text{low signal}})}{Mean_{\text{high signal}} - Mean_{\text{low signal}}}$$

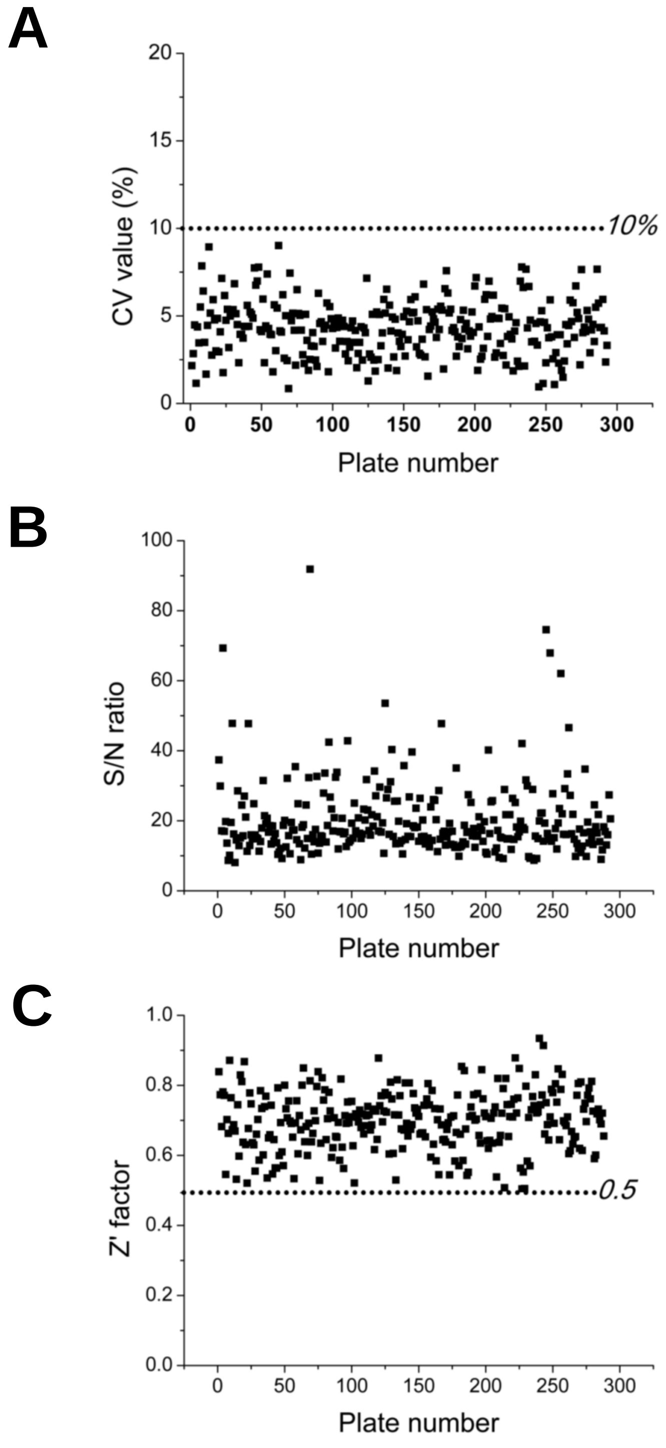

Fig. S3. NMR and Mass spectrum analysis of MS0 (735).

(A) NMR analysis of MS0 (735). (B) Mass spectrum analysis of MS0 (735).

A

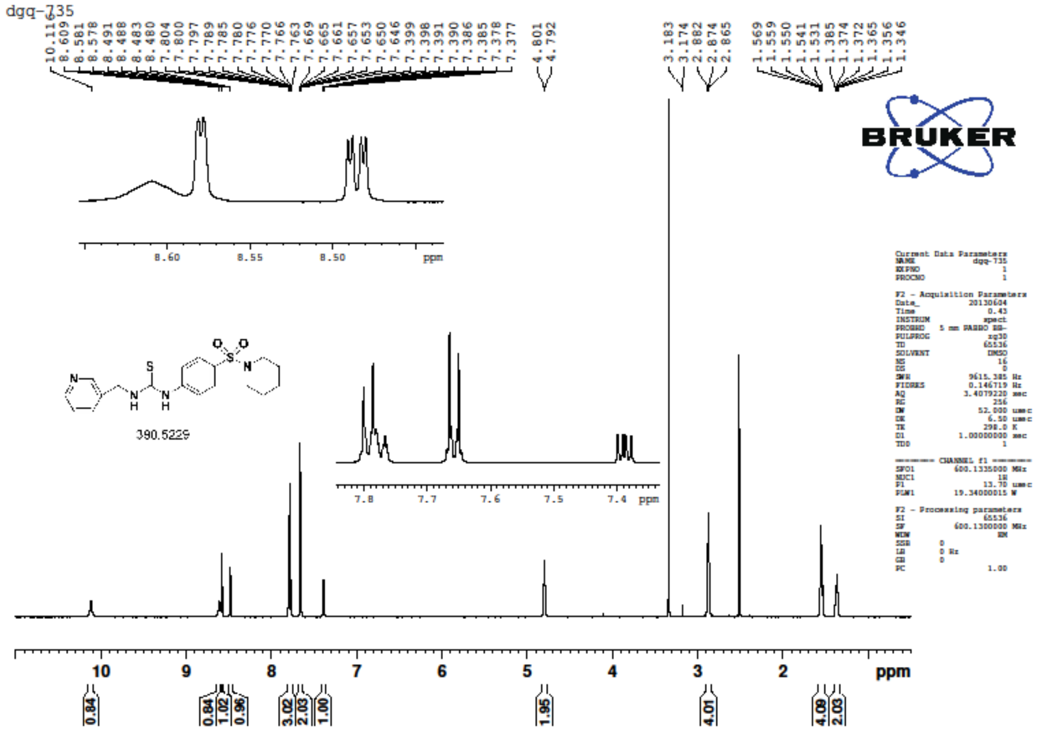

B

Qualitative Analysis Report

**Data Filename**	NAMPT735.d	**Sample Name**	NAMPT735
**Sample Type**	Sample	**Position**	P1-E7
**Instrument Name**	Instrument 1	**User Name**	
**Acq Method**	TEST-POS.m	**Acquired Time**	6/4/2013 3:43:33 PM
**IRM Calibration Status**	Some Ions Missed	**DA Method**	ERROR.m
**Comment**			

Sample Group **Info.**

**Data Filename**	NAMPT735-NEG.d	**Sample Name**	NAMPT735
**Sample Type**	Sample	**Position**	P1-E7
**Instrument Name**	Instrument 1	**User Name**	
**Acq Method**	TEST-NEG.m	**Acquired Time**	6/4/2013 3:53:36 PM
**IRM Calibration Status**	Success	**DA Method**	ERROR.m
**Comment**			

Sample Group **Info.**

User Spectra

Fragmentor Voltage	Collision Energy	Ionization Mode
120	0	Esi

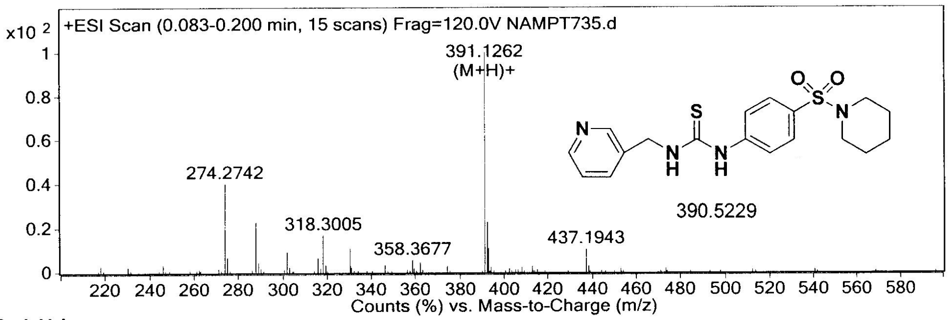

Peak List

m/z	z	Abund	Formula	Ion
274.2742	1	254576		
275.2773	1	45782		
288.2897		143763		
302.3051		60392		
318.3005		107748		
330.3365		67720		
391.1262	1	637763	C18 H23 N4 O2 S2	(M+H)+
392.1289	1	144740	C18 H23 N4 O2 S2	(M+H)+
393.1243	1	70585	C18 H23 N4 O2 S2	(M+H)+
437.1943		64453		

Formula Calculator Element Limits

Element	Min	Max
C	3	100
H	0	300
O	0	20
N	0	5
S	0	5

Formula Calculator Results

Formula	Best	Mass	Tgt Mass	Diff (ppm)	Ion Species	Score
C18 H22 N4 O2 S2	TRUE	390.1189	390.1184	-1.18	C18 H23 N4 O2 S2	99.29

Fragmentor Voltage **Collision Energy** **Ionization Mode**

Qualitative Analysis Report

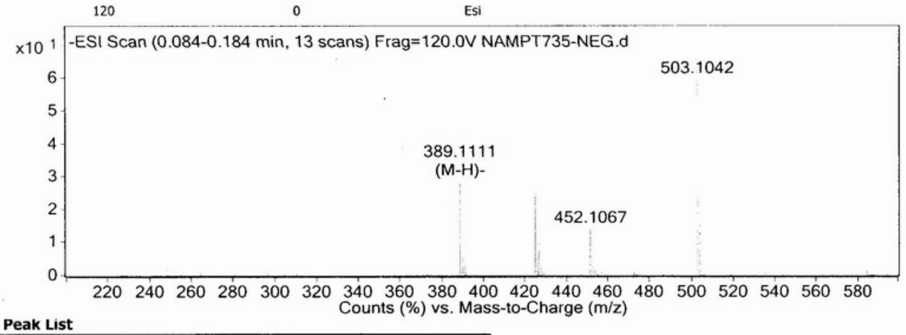

Peak List

m/z	z	Abund	Formula	Ion
112.9856		326303		
389.1111	1	184403	C18 H21 N4 O2 S2	(M-H)-
425.0877	1	160087		
427.0851	1	67431		
452.1067	1	91697		
503.1042	1	398815		
504.1068	1	96482		
505.1029	1	47510		
966.0006		46167		
1033.9881		56996		

Formula Calculator Element Limits

Element	Min	Max
C	3	100
H	0	300
O	0	20
N	0	5
S	0	5

Formula Calculator Results

Formula	Best	Mass	Tgt Mass	Diff (ppm)	Ion Species	Score
C18 H22 N4 O2 S2	TRUE	390.1184	390.1184	0.17	C18 H21 N4 O2 S2	99.57

--- End Of Report ---

Fig. S4. Identification markers of Huh7-C cells. Confocal microscopy images of Huh7-C showed some stem cell markers, including CD90, EPCAM, CD34 and CD133 (A-C). The expression of cell surface markers was also examined by fluorescence-activated cell sorting (FACS) analysis (D), and Huh7-C cells were stained with fluorescence-conjugated anti-CD90 and EPCAM. Scale bars, 20 μm (A-C).

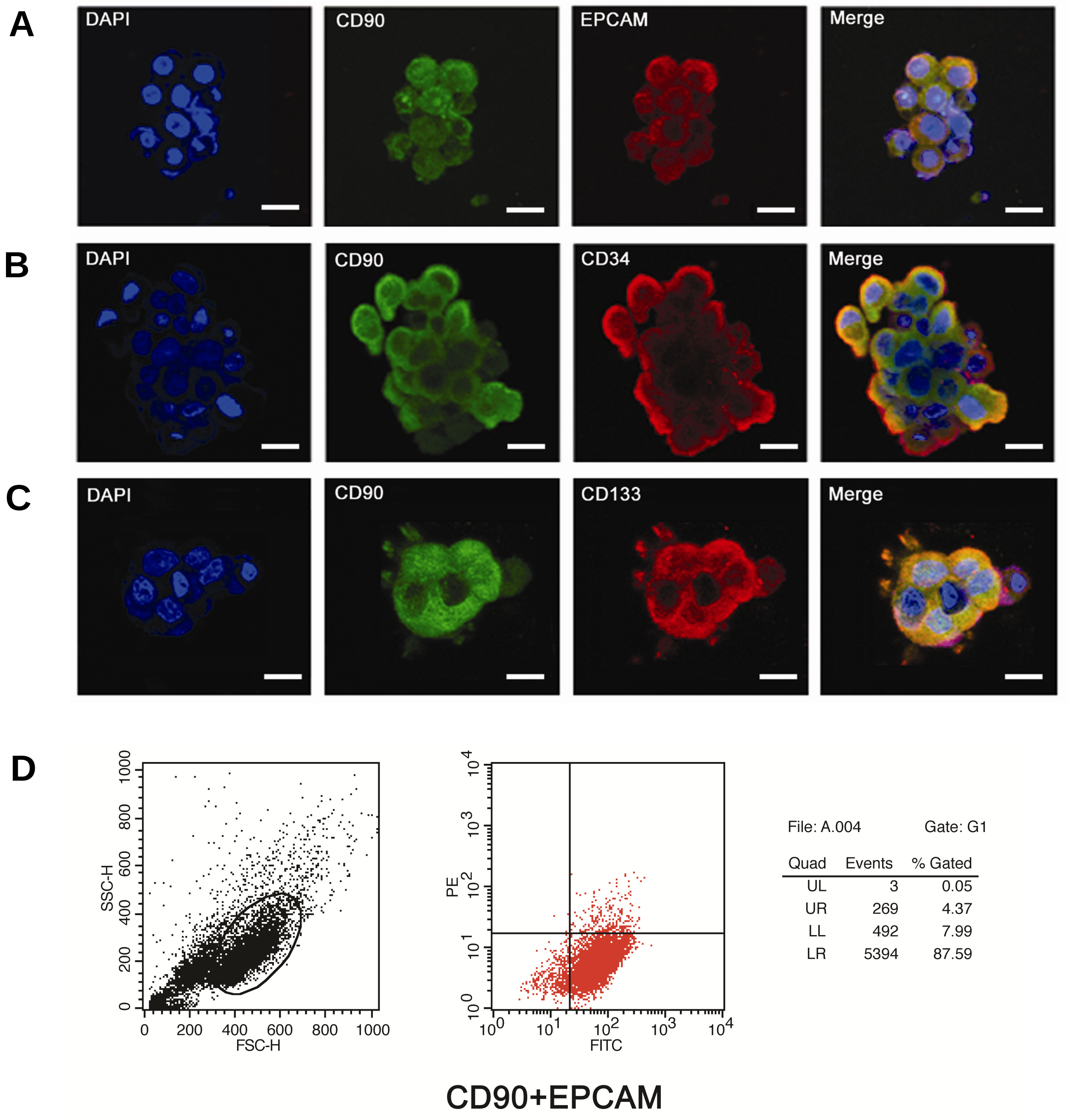

Fig. S5. Shaded area in Figure 5B and Figure 6A.

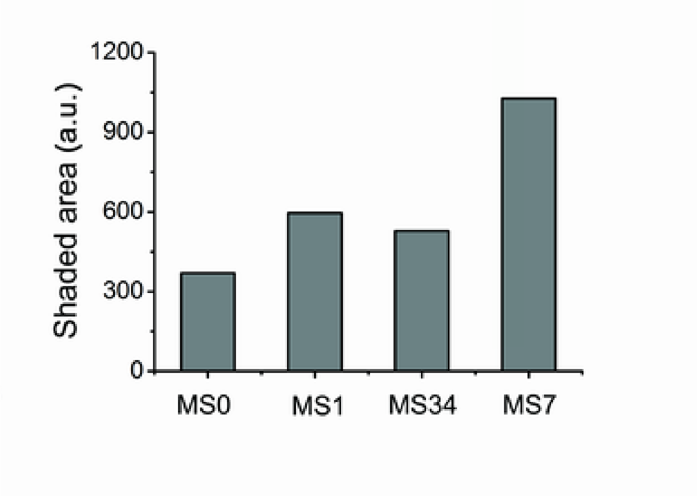

Fig. S6. Michaelis–Menten curves of wild type (WT) and mutants of NAMPT. Michaelis–Menten curves of wild type NAMPT (A) and mutants of NAMPT, H191A (B), A244S (C), S275A (D), I309Y (E) and R311M (F).

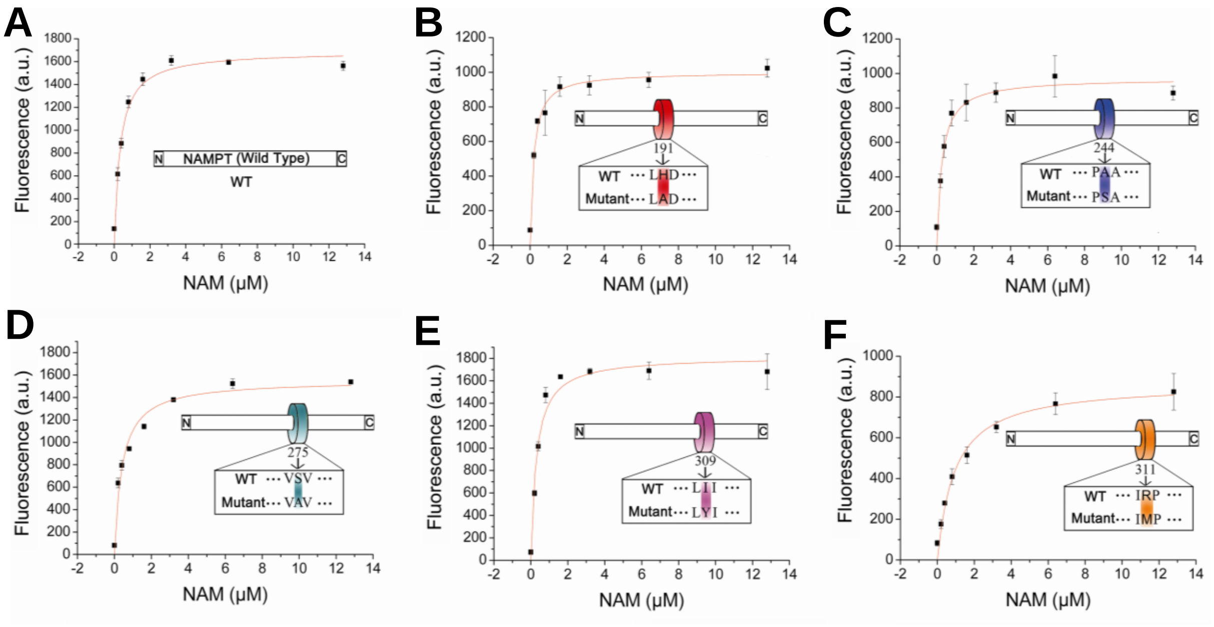

Table S1. Chemical structure, molecular weight and IC_{50} values of MS0 and its 46 analogues.

Number	Structure	Molecular weight	NAMPT IC_{50} (nM)
**MS0**	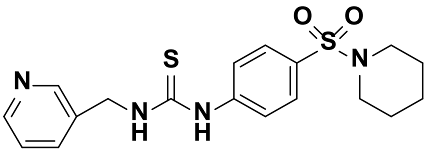	390.52	9.4±0.8
**MS1**	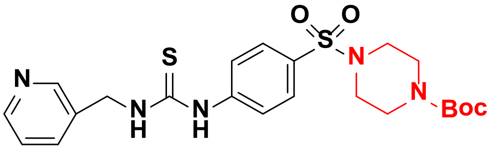	491.63	21.7±5.0
**MS2**	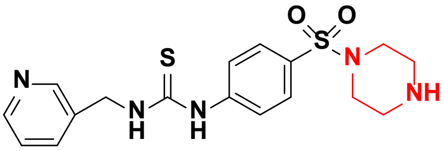	391.51	84.5±7.5
**MS3**	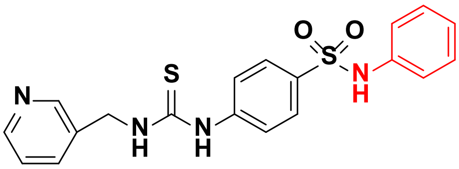	398.50	22.0±4.6
**MS4**	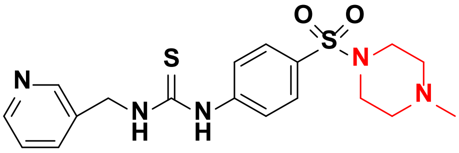	405.54	96.4±12.3
**MS5**	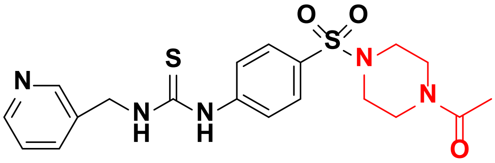	433.55	40.5±1.8
**MS6**	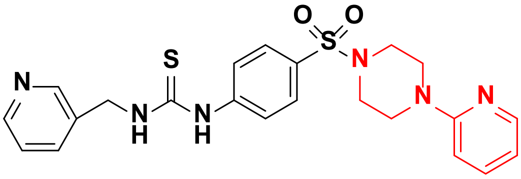	468.59	17.7±3.4
**MS7**	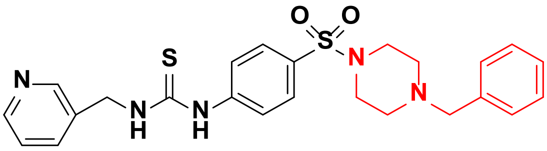	481.63	0.9±0.3

**MS8**	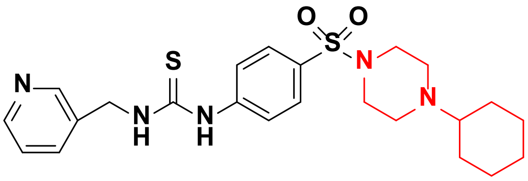	473.65	14.2±2.4
**MS9**	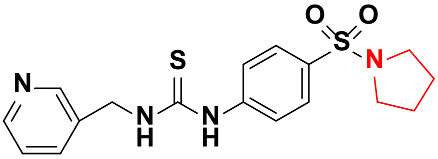	376.50	43.9±2.4
**MS10**	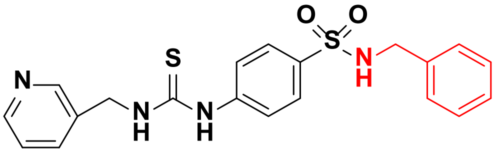	412.53	19.6±0.2
**MS11**	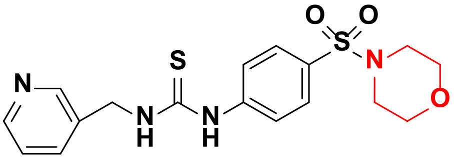	392.50	21.7±0.8
**MS12**	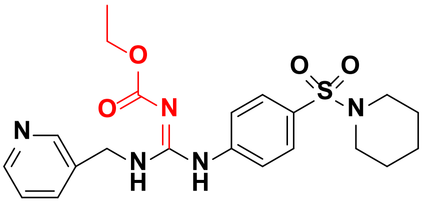	445.54	>166 µM
**MS13**	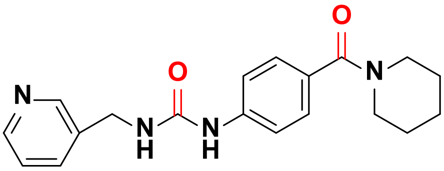	338.40	98.9±10.3
**MS14**	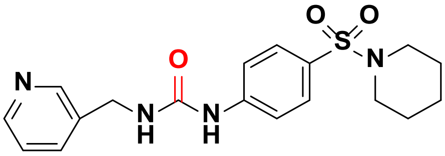	374.46	20.1±0.8
**MS15**	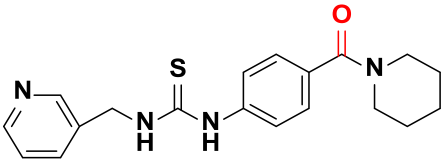	354.47	42.2±3.1
**MS16**	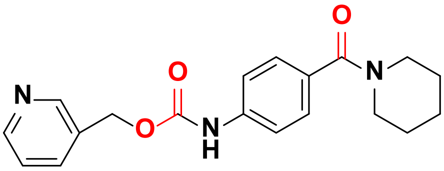	339.39	52.4±6.2
**MS17**	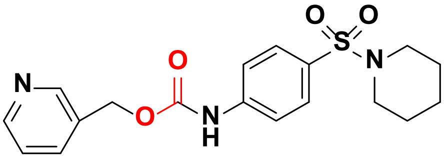	375.44	42.8±4.0

**MS18**	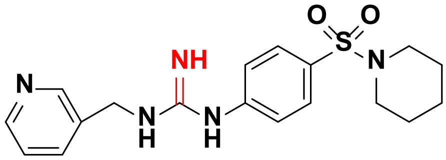	373.47	765.0±74.8
**MS19**	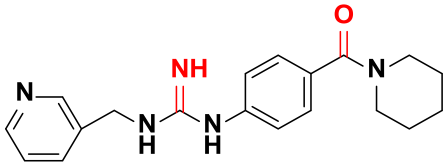	337.42	14533.3±202.8
**MS20**	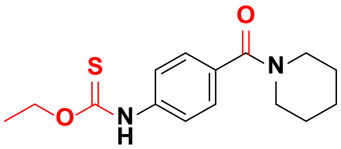	292.40	>150 µM
**MS21**	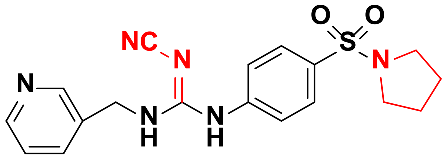	384.46	577.1±38.7
**MS22**	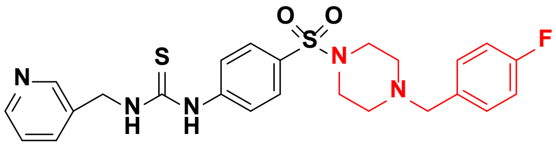	499.62	12.0±1.5
**MS23**	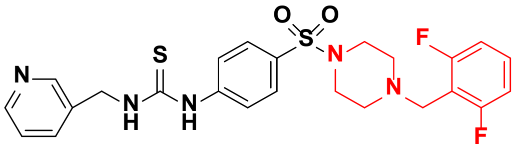	517.61	9.9±1.2
**MS24**	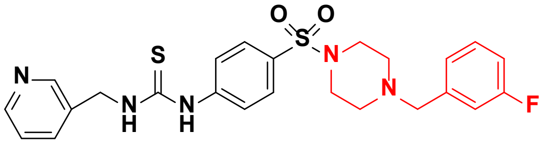	499.62	8.9±0.5
**MS25**	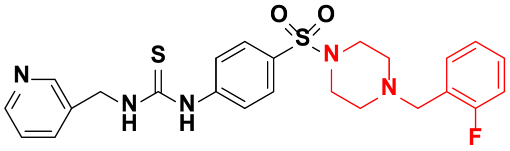	499.62	12.3±2.0
**MS26**	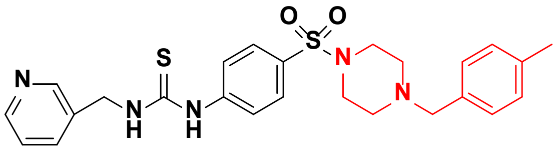	495.66	10.5±2.0
**MS27**	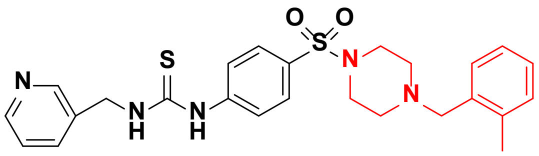	495.66	12.4±0.9

**MS28**	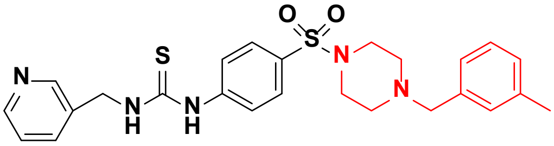	495.66	16.8±1.5
**MS29**	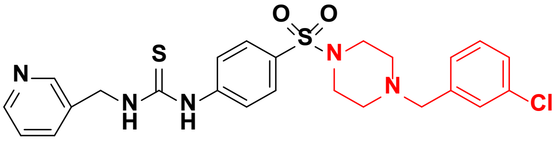	516.08	12.6±1.7
**MS30**	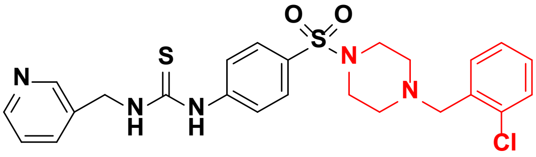	516.08	15.7±2.5
**MS31**	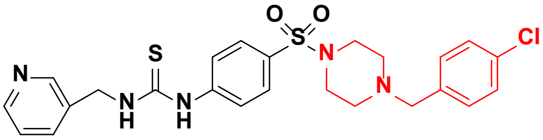	516.08	13.7±1.6
**MS32**	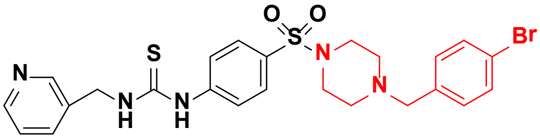	560.53	24.2±1.6
**MS33**	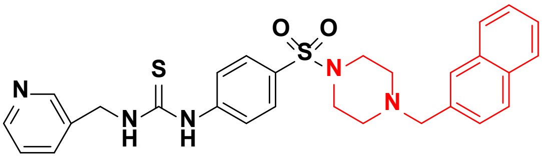	531.69	29.5±0.8
**MS34**	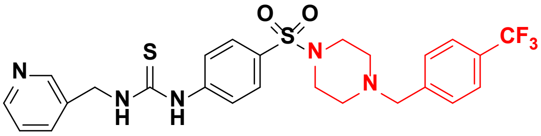	549.63	23.7±7.8
**MS35**	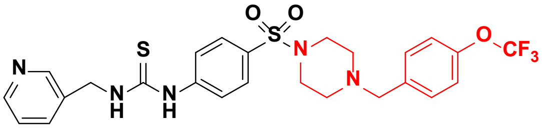	565.63	36.9±4.6
**MS36**	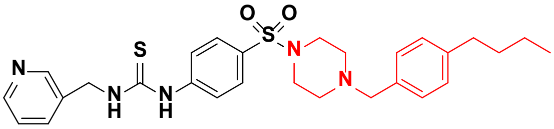	537.74	79.8±6.1

**MS37**	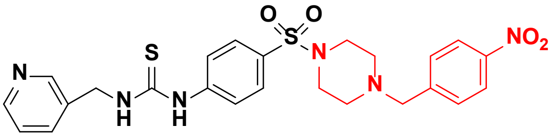	526.63	19.7±2.1
**MS38**	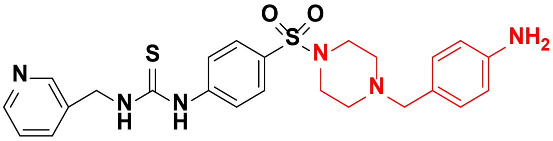	496.65	19.9±2.8
**MS39**	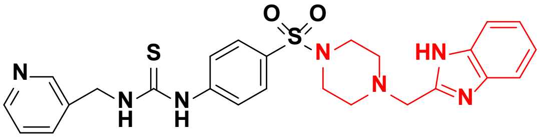	521.66	14.0±0.3
**MS40**	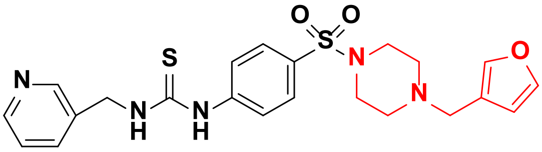	471.60	48.7±4.5
**MS41**	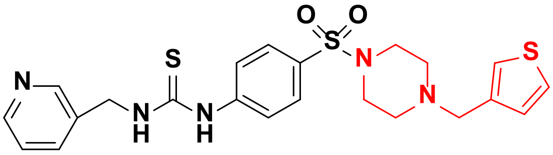	487.66	22.5±0.8
**MS42**	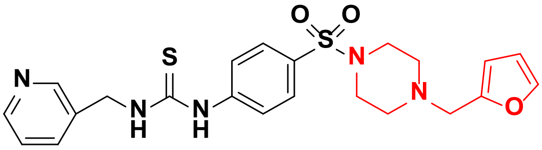	471.60	19.1±2.0
**MS43**	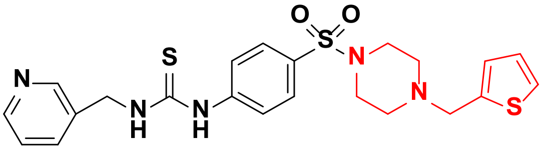	487.66	38.8±1.8
**MS44**	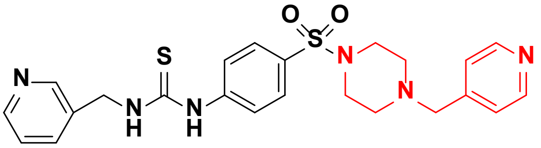	482.62	13.7±0.4

**MS45**	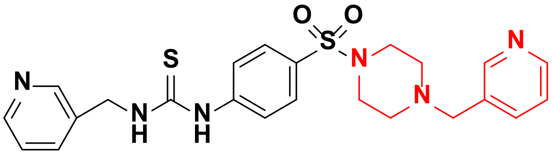	482.62	9.8±0.9
**MS46**	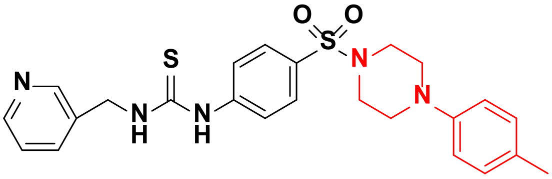	481.63	15.4±1.6

Supplemental Materials and Methods

Part Ⅰ

Chemicals

A chemical library used for high throughput screening contains 24434 small-molecules, including 9234 from the National Compound Resource Center (Shanghai, China), 14400 from Maybridge and 800 from an in-house compound collection. Compounds MS0 and 46 novel analogues were synthesized by our research group. All synthesized chemicals were dissolved and diluted using dimethyl sulfoxide (DMSO) except special announcement.

Plasmids construction of NAMPT wild-type and mutants

cDNA sequence of human NAMPT was amplified by PCR from pGex-6p-3-hNAMPT plasmid (kindly gift from Dr. Shui-Qing Ye in University of Missouri) using the following primers: forward, 5’-GGACATATGATGAATCCTGCGGCAGAAGC-3’; and reverse, 5’-AATCTCGA -GGTAATGATGTGCTGCTTCCAGTTC-3’. The PCR products were digested and cloned into pET21a+ vector using NdeI and XhoI restriction enzyme. Point mutation was introduced by quick change site-directed mutagenesis method[1] using the constructed pET21a+-hNAMPT plasmid as a template. Primers used for mutagenesis were as follows: H191A forward, 5’-CTGGAATACAAGTTAGCTGATTTTGGCTACAGAG-3', and reverse, 5'-CTCTGTAGCCAAAATCAGCTAACTTGTATTCCAG-3'; A244S forward: 5'-GCTATTCTG -TTCCATCAGCAGAACACAG-3', and reverse: 5'-GTACTGTGTTCTGCTAGTGGAACAGA -ATAGCC-3'; S275A forward: 5'-CATCAGTGCCTGTAGCTGTGGTCAGCG 3', and reverse: 5'-CGCTGACCACAGCTACAGGCACTGATG-3'; I309Y forward 5'-CACAGGCACCACTATA -CATCAGACCTGATTCTGG-3', and reverse: 5'-CCAGAATCAGGTCTGATGTATAGTGGTG -CCTGTG-3'; R311M forward: 5'-GCACCACTAATAATCATGCCTGATTCTGGAAAC-3', and reverse: 5'-GTTTCCAGAATCAGGCATGATTATTAGTGGTGC-3’. All the mutations were validated by DNA sequencing.

Protein expression and purification

Proteins were expressed and purified by our previous methods[2,3]. His-tagged NAMPT wild-type, NAMPT mutants and NMNAT1 (mouse NMNAT1-pET28a+ plasmid is a kind gift from Dr. Shin-ichiro Imai in Washington University) were expressed in BL21-CondonPlus (DE3)-RIL cells (Stratagene) at 28 °C, in 2×YT media containing 100 μg/ml kanamycin and 37 μg/ml chloramphenicol, and then purified with nickel-nitrilotriacetic acid resin (Qiagen). The purity of the protein was more than 90% determined by SDS-PAGE and Coomassie Blue (Tiangen, China) staining and further by western blot analysis (Fig. S1).

High throughput screening (HTS)

HTS was performed using our previously reported method [3]. 0.5 μl stock of each compound (1 mM DMSO stock) was transferred to a 96-well PCR plate for screening. In the primary screening, 5 ng NAMPT in 20 μl reaction buffer [0.4 mM phosphoribosylpyrophosphate (PRPP, Sigma), 2 mM ATP, 0.02% BSA, 2 mM DTT, 12 mM $MgCl_2$ and 50 mM Tris-HCl (pH = 7.5)] was added into each well, the plate was incubated at 37°C for 5 min, then 4.5 μl substrate of NAM was added to initiate the enzyme reaction, resulting in a final concentration of 2% DMSO, 2 μg/ml NAMPT, 0.2 μM NAM and 20 μM compound. After reacting at 37°C for 15 min, the enzyme reaction was terminated by heating at 95 °C for 1 min and cooling in an ice bath. The product of NMN was detected through the following approach: after adding 10 μl 20% acetophenone in DMSO and 10 μl 2 M KOH into each well, the mixture was vortex-mixed and kept in ice bath for 2 min. Then 45 μl 88% formic acid was added and the mixture was incubated at 37°C for 10 min. Finally, 85 μl mixtures in each well were transferred into a flat-bottom 96-well black plate (Greiner), and the fluorescence (F) was measured using a Tecan Infinity M200 plate reader (Tecan Group Ltd.) by setting the excitation and emission wavelength to 382 nm and 445nm respectively. The last row of each 96-well plate includes six wells of background controls and six wells of reference controls. The relative enzyme activity (Activity%) regulated by specific compound was calculated according to equation (1):

$$\text{Activity\%} = \frac{F - F_0}{F_{100\%} - F_0} \quad (1)$$

F_0 was the averaged fluorescence of six background controls, representing zero activity from a simulated enzyme reaction with only NAMPT but no NAM and compound; $F_{100\%}$ was the averaged fluorescence of six reference controls, representing 100% activity from intact enzyme reaction without compound perturbation.

Compounds with Activity% less than 40% were considered as inhibitors and subjected to a secondary screen inhibition validation, in which another background control (F_{C0}), a simulated enzyme reaction with NAMPT and compound but no NAM, was introduced to eliminate the direct and/or indirect influence from compound. At this stage, the Activity% was calculated according to equation (2):

$$\text{Activity\%} = \frac{F - F_{C0}}{F_{100\%} - F_0} \qquad (2)$$

In the screening, the signal-to-noise (S/N) ratio was calculated using the equation: ($\text{Mean}_{\text{signal}} - \text{Mean}_{\text{background}}$) / $\text{SD}_{\text{background}}$[4]. Coefficients of variation (CV) were the ratio of SD to mean. The Z' factor was determined by equation (3)[5]:

$$Z' = 1 - \frac{3(\text{SD}_{\text{high signal}} + \text{SD}_{\text{low signal}})}{\text{Mean}_{\text{high signal}} - \text{Mean}_{\text{low signal}}} \qquad (3)$$

Determination of IC_{50} for NAMPT inhibitors

To determine the IC_{50} of inhibitors, 5 μl compound solutions (containing 10% DMSO) with various concentrations were added into 96-well plate. The plate was incubated at 37 °C for 5 min after addition of 16.5 μl reaction buffer containing NAMPT. The enzyme reactions were initiated by 4.5 μl NAM (1.11 μM) following NMN measurement as described above. The IC_{50} values were determined by non-linear fitting of the concentration-dependent curves with the four-parameter IC_{50} logistic equation.

NAD measurement

Cellular level of NAD was measured by spectrophotometric enzymatic cycling assay, as described previously[6-8]. Briefly, cells were seeded in 96-well plate and starved for over 12 h with serum-free DMEM at 60~70% confluency, following by treatment with compounds or vehicle for 24 h. Cells were lysed with 50 μl of 1M $HClO_4$ on ice for 30 min. The lysates

were cleared by centrifuging at 4 °C at 18,000×g for 5 min, and cleared lysates (40 μl) were neutralized by adding 1M K_2CO_3 (16 μl) and incubation on ice for 20 min. After centrifuging for 10 min, 10 μl of supernatant were mixed with reaction buffer [50 mM Tris-HCl (pH 7.5), 3% ethanol, 1.66 mM PES (phenazineethosulfate), 0.42 mM MTT (3-(4,5-dimethylthiazolyl-2)-2,5-diphenyltetrazolium bromide), 90 μg/ml ADH] in a total volume of 100 ul, and incubated at 37 °C for 40 min. The absorbance at 570 nm was determined. A blank measurement without ADH was also carried out. Each experiment was carried out in triplicate in three replicate wells.

Cell counting kit-8 (CCK-8) assay

Cell viability was determined by our previous method[9] using the Cell Counting Kit-8 (CCK-8, Dojindo, Japan). In human hepatocellular carcinoma cell line HepG2, cells were seeded in 96-well plate and starved for over 12 h with serum-free DMEM at 60~70% confluency, then treated with compounds or vehicle for 24 h to 72 h according to experiment requirements. In stem like Huh7-C cells, cells were seeded in 96-well plate (10^4 cells per well), and treated with compounds or vehicle for 60 h. 10 μl CCK-8 solution was added to the culture medium and incubated at 37 °C for 1 h. The absorbance at 450 nm (A450) was detected by a plate reader. Each experiment was carried out in triplicate in three replicate wells.

Sulforhodamine B protein staining (SRB) assay

Cell proliferation was evaluated by sulforhodamine B protein staining (SRB) assay[10-12]. Briefly, cells were seeded into 96-well plates, cultured overnight, and treated with corresponding compounds for 72 h. Cells were then fixed with 10% trichloroacetic acid and stained with sulforhodamine B (Sigma). Sulforhodamine B in the cells was dissolved in 10 mmol/L Tris-HCl and was measured at 515 nm using a multiwell spectrophotometer (VERSAmax, Molecular Devices, Sunnyvale, CA). The inhibition rate on cell proliferation was calculated for each well as $(A515_{control\ cells} - A515_{treated\ cells})/A515_{control\ cells} \times 100\%$ (A515: OD value at 515 nm). The average IC_{50} values were determined by Logit method from at least three independent tests.

Cell culture and in vitro experiments

Human hepatocellular carcinoma cell line HepG2, human osteosarcoma cell line U2OS and human lung adenocarcinoma cell line A549 were purchased from the American Type Culture Collection (Manassas, VA, USA). Human colorectal carcinoma cell line HCT-116 was from the Japanese Foundation of Cancer Research (Tokyo, Japan). Human ovarian cancer cell line A2780 and human invasive lung cancer cell line 95-D were from Cell Bank of Chinese Academy of Sciences (Shanghai, China). Hepatocellular carcinoma stem-like cells Huh7-C was from Laboratory of Viral and Gene Therapy, Eastern Hepatobiliary Surgical Hospital & Institute, Second Military Medical University. HepG2, A2780, U2OS were maintained in Dulbecco's Modified Eagle Medium (GIBCO, Grand Island, NY, USA). A549, HCT-116 and 95-D were cultured in RPMI-1640 medium (GIBCO). Both culture media were supplemented with 10% heat-inactivated fetal bovine serum (GIBCO, Grand Island, NY, USA), L-glutamine (2 mmol/L), penicillin (100 IU/mL), streptomycin (100 μg/mL). Huh7-C cells were cultured in the system which contains ingredients 50% DMEM/F12, 50% Neurobasal™ - A Medium, 1% B27 without VA, 1% GlutaMAX™ - I, 1% NEAA, 20 ng/mL EGF, 4 μg/mL Heparin, 0.1 mM 2-Mercaptoethanol, 0.4% BSA, and 2 cytokines as 20 ng/mL FGF-10 and 20 ng/mL IGF-1. All cell lines were cultured in a humidified atmosphere of 95% air plus 5% CO_2 at 37 ℃, as our previously reported [8,13].

Isothermal titration calorimetry (ITC)

Thermodynamic parameters of small molecule binding to protein were determined using a MicroCal VP-ITC calorimeter[14]. NMNAT protein solutions were ultrafiltrated in Amicon Ultra-0.5 Centrifugal Filter Unit (Merck Millipore) against buffer [20 mM Tris (pH 7.5), 20 mM NaCl], which was subsequently used to prepare a matched compound solution. ITC data collected for MS0 were acquired in 5% DMSO to improve compound solubility. Each isotherm was recorded by injecting 554 μM MS0 into 25 μM solutions of protein. Measurements were performed at 25°C with spacing of 90 s between injections. Background signal (calculated as a mean value) generated by addition of MS0 to buffer was subtracted prior to analysis on Origin 7.5 using software supplied by the manufacturer.

Cellular thermal shift assay (CETSA)

CESTA was performed as previously described[15]. In the cell lysate CETSA experiments, cultured cells were harvested and washed with PBS. All buffers were supplemented with complete protease inhibitor cocktail. The cells were diluted in PBS. The cell suspensions were freeze-thawed three times using liquid nitrogen. The soluble fraction (lysate) was separated from the cell debris by centrifugation at 20000 x g for 20 min at 4°C. The cell lysates were diluted with PBS and divided into two aliquots, with one aliquot being treated with drug and the other aliquot with the diluent for the corresponding drug (control). MS0, MS1, MS7 and MS34 were added from DMSO stocks to the final concentration of 100 µM and DMSO concentration 1% except those in $ITDRF_{CESTA}$. Control samples were incubated with an equal amount of DMSO. After 30 min incubation at room temperature the respective lysates were divided into smaller (50 µL) aliquots and heated individually at different temperatures for 3 min (Veriti thermal cycler, Applied Biosystems/Life Technologies) followed by cooling for 3 min at room temperature. The appropriate temperatures were determined in preliminary CETSA experiments (data not shown). The heated lysates were centrifuged at 20000 x g for 20 min at 4°C in order to separate the soluble fractions from precipitates. The supernatants were transferred to new microtubes and analyzed by sodium dodecyl sulfate polyacrylamide gel electrophoresis (SDS-PAGE) followed by Western blot analysis.

In the intact cell experiments, treated cells were exposed to a drug for 3 h in cell culture incubator (95% O_2 and 5% CO_2). Control cells were incubated with an equal volume of diluent for the corresponding drug. MS0, MS1, MS7 and MS34 were added from DMSO stocks to the final concentration of 10 µM and DMSO concentration 0.1% except those in $ITDRF_{CESTA}$. Following the incubation the cells were harvested using Trypsin/EDTA solution and washed with PBS in order to remove excess drug. Equal amounts of cell suspensions were aliquoted into 0.2 mL PCR microtubes, and excess PBS was removed by centrifugation to leave 10 uL or less PBS in each microtube. These cell pellets were heated as previously described and lysed using 2 cycles of freeze-thawing with liquid nitrogen. The soluble fractions were isolated and analyzed by Western blot analysis as described above.

SDS-PAGE and Western blot analysis

Target proteins in CESTA were examined by SDS-PAGE and immunoblotting as previously described[8,13,15,16]. The primary antibodies for Western blotting were specific for the following: NAMPT (Santa Cruz, sc-67020, used at 1:500), HDAC1 (Cell Signaling, 5356, used at 1:300), and HDAC3 (Cell Signaling, 3949, used at 1:300). To determine the effect of MS0, MS1, MS7 and MS34 on acetylation of histone H3 at Lys9 and Lys18, total cellular and nuclear proteins were extracted according to the instructions of the nuclear and cytoplasmic protein extraction kit (Beyotime, Haimen, China). The protein content of the nuclear extracts was estimated using an enhanced BCA protein assay kit (according to the manufacturer's instructions). Fifty micrograms of protein from each sample were subjected to SDS-PAGE. The nuclear extracts were separated by SDS-PAGE. The primary antibodies used were: anti-H3K9 (Cell Signaling, 9753, used at 1:3000), anti-H3K18 (Cell Signaling, 9759, used at 1:3000), and anti-H3-total (Abcam, ab1791, used at 1:3000). Secondary antibodies were IRDye800CW goat anti-rabbit IgG and IRDye700CW goat anti-mouse IgG (LI-COR Biosciences, Nebraska, USA). The images were captured and analyzed by the Odyssey infrared fluorescence imaging system (Li-Cor Bioscience). Each experiment was repeated at least three times.

Binding mode study of MS0 with NAMPT

AutoDock_Vina1.1.2 program was used to build the binding mode of MS0 with NAMPT. The 3D-structure of MS0 was modeled and energy-minimized in Chem3D program, and the coordinates of NAMPT (PDBID: 2GVJ) were retrieved from the Protein Data Bank website. Both structures of MS0 and NAMPT were pre-processed in AutoDockTools1.5.4[17], such as merge non-polar hydrogens, add Gasteiger charges, set rotatable bond for MS0, add solvation parameter, and so on. The docking space of $15 \times 15 \times 30 Å^3$ was visually set around the binding site of FK866, the parameter of exhaustiveness, num modes and energy range was set to 20, 1000 and 5 respectively, and the default values were used for the other parameters.

Statistical analysis

Data are expressed as the mean ± s.e. Statistical comparisons between two groups were

performed by Student’s t test. Comparisons among several groups (≥ 3 groups) were performed by analysis of variance followed by Tukey’s post hoc test. Statistical significance was set at $P < 0.05$.

Part Ⅱ

Chemical Synthesis:

Scheme 1

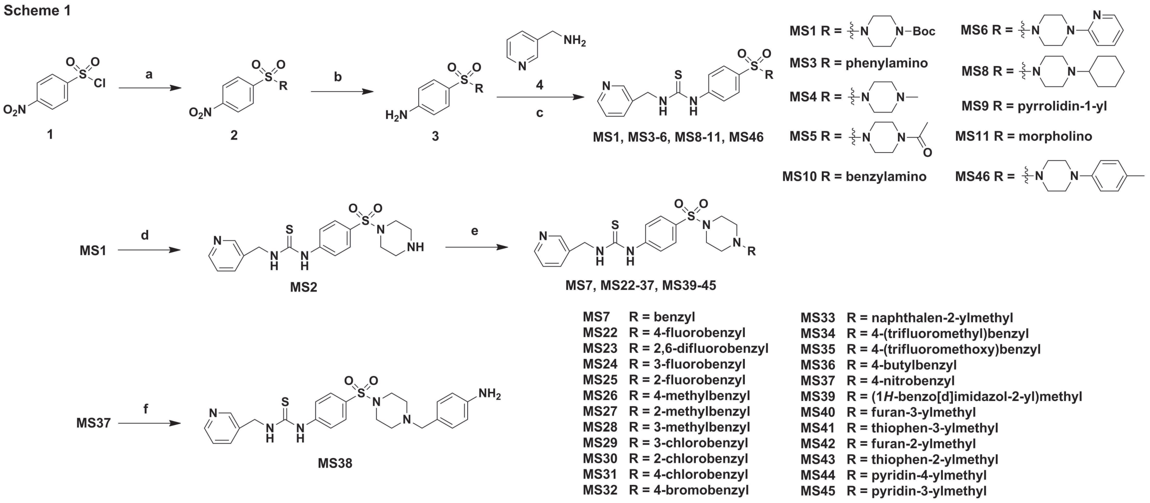

Reagents and conditions: (a) CH_2Cl_2, RT, 2 h, yield 100%; (b)Pd/C, H_2, EtOAc, RT, 12 h, yield 50-72%; (c) thiophosgene, $NaHCO_3$, CH_2Cl_2, C_2H_5OH,RT, 1 h, yield 33-51%; (d) TFA, CH_2Cl_2, RT,1 h, yield 75%; (e) RX, Et_3N, acetone, RT, 1 h, yield 31-76%; (f) Pd/C, H_2, EtOAc, RT, 12 h, yield 72%;

Experimental section

Chemistry. General Methods. Nuclear magnetic resonance (NMR) spectra were recorded on a Bruker AVANCE300 and AVANCE500 spectrometer (Bruker Company, Germany), with TMS as an internal standard and d_6-DMSO as the solvent. Chemical shifts (δ values) and coupling constants (*J* values) are expressed in ppm and Hz, respectively. ESI mass spectra were performed on an API-3000 LC–MS spectrometer. TLC analysis was carried out on silica gel plates GF254 (QindaoHaiyang Chemical, China). Silica gel column chromatography was performed with Silica gel 60 G (QindaoHaiyang Chemical, China). Commercial solvents were used without any pretreatment.

***tert*-butyl 4-((4-(3-(pyridin-3-ylmethyl)thioureido)phenyl)sulfonyl)piperazine-1-carboxylate (MS1).** A solution of sodium bicarbonate (0.17 g, 2 mmol) in water (20 mL) was stirred for 10 min and to it was added CH_2Cl_2 (20 mL) followed by *tert*-butyl 4-((4-aminophenyl)sulfonyl)piperazine-1-carboxylate[18] (**3a**, 0.34 g, 1 mmol). Thiophosgene (0.14 g, 1.2 mmol) was added dropwise over a period of 30 min and the reaction mixture was continuously stirred at room temperature for 1 h. The reaction mixture was washed with brine

solution (20 mL); the organic layer was dried over anhydrous Na_2SO_4 and concentrated to get a crude orange compound. The orange compound was dissolved in C_2H_5OH (20 mL) and thenpyridin-3-ylmethanamine (0.11 g, 1 mmol) was added. After stirred for 1 h at room temperature, the solvent was removed under reduced pressure. The residue was purified by silica gel column chromatography (CH_2Cl_2 : MeOH = 100:2) to afford **MS1** as a white solid (0.21 g, yield 41%). ^{1}H NMR (*d*-DMSO, 600 MHz) δ: 1.34 (s, 9H), 2.83-2.85 (m, 4H), 3.37-3.39 (m, 4H), 4.78 (s, 2H), 7.37 (dd, *J* = 7.8 Hz, 4.8 Hz, 1H), 7.65 (d, *J* = 9.0 Hz, 2H), 7.76 (d, *J* = 7.8 Hz, 1H), 7.80 (d, *J* = 9.0 Hz, 2H), 8.47 (dd, *J* = 4.8 Hz, 1.2 Hz, 1H), 8.57 (d, *J* = 1.8 Hz, 1H), 8.61 (s, 1H), 10.11 (s, 1H). ESI-HRMS (m/z): 492.1722 [M+1].

The synthetic method for target compounds **MS0**, **MS3-6**, **MS8-11**, **MS15**, **MS46** was similar to the synthesis of compound **MS1**.

1-(4-(piperazin-1-ylsulfonyl)phenyl)-3-(pyridin-3-ylmethyl)thiourea (MS2). A solution of **MS1** (0.10 g, 0.2mmol) in CH_2Cl_2/TFA (2:1, 10 mL) was stirred at room temperature for 1 h. The solvent was removed under reduced pressure and the residue was diluted with saturated sodium bicarbonate (50 mL) and then extracted with EtOAc (3× 50 mL). The combined organic layers were washed with saturated sodium chloride solution (3 ×50mL), dried over anhydrous Na_2SO_4 and concentrated under reduced pressure. The residue was purified by silica gel column chromatography (gradient CH_2Cl_2 : MeOH = 100:1 to 100:5) to give compound **MS2** as a white solid (0.06 g, yield 75%). ^{1}H NMR (*d*-DMSO, 600 MHz) δ: 2.70-2.73 (m, 8H), 4.76 (s, 2H), 7.36 (dd, *J* = 7.2 Hz, 4.8 Hz, 1H), 7.60 (d, *J* = 8.7 Hz, 2H), 7.75 (d, *J* = 7.8 Hz, 1H), 7.85 (d, *J* = 8.7 Hz, 2H), 8.46 (m, 1H), 8.55 (s, 1H), 9.20 (s, 1H), 10.75 (s, 1H). ESI-MS (m/z): 392.12 [M+1].

***N*-phenyl-4-(3-(pyridin-3-ylmethyl)thioureido)benzenesulfonamide (MS3).** White solid (0.16 g, yield 39%). ^{1}H NMR (*d*-DMSO, 600 MHz) δ: 4.75 (d, *J* = 5.3 Hz, 2H), 7.00 (t, *J* = 7.3 Hz, 1H), 7.10 (d, *J* = 7.8 Hz, 2H), 7.22 (t, *J* = 7.8 Hz, 2H), 7.35 (dd, *J* = 7.8 Hz, 4.6 Hz, 1H), 7.66-7.69 (m,4H), 7.74 (d, *J* = 7.8 Hz, 1H), 8.46 (dd, *J* = 4.8 Hz, 1.7 Hz, 1H), 8.54-8.55 (m, 2H), 9.98 (s, 1H), 10.20 (s, 1H). ESI-MS (m/z): 399.61 [M+1].

1-(4-((4-methylpiperazin-1-yl)sulfonyl)phenyl)-3-(pyridin-3-ylmethyl)thiourea (MS4). White solid (0.18 g, yield 45%). ^{1}H NMR (*d*-DMSO, 600 MHz) δ: 2.14 (s, 3H), 2.35-2.36 (m, 4H), 2.87-2.88 (m, 4H), 4.79 (s, 2H), 7.37 (dd, *J* = 7.8 Hz, 4.7 Hz, 1H), 7.65 (d, *J* = 8.8 Hz, 2H), 7.77 (d, *J* = 7.8 Hz, 1H), 7.79 (d, *J* = 8.8 Hz, 2H), 8.47 (dd, *J* = 4.7 Hz, 1.2 Hz, 1H), 8.57 (s, 1H), 8.58 (s, 1H), 10.05 (s, 1H). ESI-MS (m/z): 406.62 [M+1].

1-(4-((4-acetylpiperazin-1-yl)sulfonyl)phenyl)-3-(pyridin-3-ylmethyl)thiourea (MS5). White solid(0.14 g, yield 33%). ^{1}H NMR (*d*-DMSO, 600 MHz) δ: 1.93 (s, 3H), 2.85-2.91 (m, 4H), 3.48-3.51 (m, 4H), 4.79 (d, *J* = 5.4 Hz, 2H), 7.37 (dd, *J* = 7.8 Hz, 4.7 Hz, 1H), 7.66 (d, *J* = 8.8 Hz, 2H), 7.76 (d, *J* = 7.8 Hz, 1H), 7.81 (d, *J* = 8.8 Hz, 2H), 8.48 (d, *J* = 4.7 Hz, 1H), 8.57 (br s, 2H), 10.06 (s, 1H). ESI-MS (m/z): 434.44 [M+1].

1-(4-((4-(pyridin-2-yl)piperazin-1-yl)sulfonyl)phenyl)-3-(pyridin-3-ylmethyl)thiourea (MS6). White solid (0.23 g, yield 50%). ^{1}H NMR (*d*-DMSO, 600 MHz) δ: 2.96 (t, *J* = 5.1 Hz, 4H), 3.58 (t, *J* = 5.1 Hz, 4H), 4.77 (d, *J* = 5.6 Hz, 2H), 6.63 (dd, *J* = 7.1 Hz, 5.0 Hz, 1H), 6.80 (d, *J* = 8.6 Hz, 1H), 7.35-7.37 (m, 1H), 7.49-7.52 (m, 1H), 7.68 (d, *J* = 8.8 Hz, 2H), 7.75 (d, *J* = 7.7 Hz, 1H), 7.79 (d, *J* = 8.8 Hz, 2H), 8.06-8.08 (m, 1H), 8.46 (dd, *J* = 4.8 Hz, 1.7 Hz, 1H), 8.56 (d, *J* = 1.7 Hz, 1H), 8.59 (m, 1H), 10.08 (s, 1H). ESI-MS (m/z): 469.42 [M+1].

1-(4-((4-benzylpiperazin-1-yl)sulfonyl)phenyl)-3-(pyridin-3-ylmethyl)thiourea (MS7). To a stirring solution of **MS2** (0.1 g, 0.25 mmol) and Et_3N (0.05 mL, 0.38 mmol) in acetone (20 mL), benzyl bromide (35μL, 0.3mmol) was added and stirred for 1h at room temperature. Then solvent was removed under reduced pressure. The residue was purified by silica gel column chromatography (gradient CH_2Cl_2 : MeOH = 100:1 to 100:3) to afford **MS7** as a white solid (0.20 g, yield 42%). ^{1}H NMR (*d*-DMSO, 600 MHz) δ: 2.42-2.43 (m, 4H), 2.89-2.91 (m, 4H), 3.46 (s, 1H), 4.80 (d, *J* = 6.0 Hz, 2H), 7.21-7.23 (m, 3H), 7.26-7.29 (m, 2H), 7.37 (dd, *J* = 7.7 Hz, 4.7 Hz, 1H), 7.64 (d, *J* = 8.9 Hz, 2H), 7.76 (d, *J* = 7.7 Hz, 1H), 7.80 (d, *J* = 8.9 Hz, 2H), 8.48 (dd, *J* = 4.7 Hz, 1.3 Hz, 1H), 8.58 (br s, 2H), 10.05 (s, 1H). ESI-HRMS (m/z): 482.1678 [M+1].

The synthetic method for target compounds **MS22-37**, **MS39-45** was similar to the synthesis of compound **MS7**.

1-(4-((4-cyclohexylpiperazin-1-yl)sulfonyl)phenyl)-3-(pyridin-3-ylmethyl)thiourea (MS8). White solid (0.21 g, yield 44%). ^{1}H NMR (*d*-DMSO, 600 MHz) δ: 1.02-1.18 (m, 6H), 1.67-1.68 (m, 4H), 2.20 (s, 1H), 2.53-2.54 (m, 4H), 2.84-2.85 (m, 4H), 4.79 (d, *J* = 5.6 Hz, 2H), 7.37 (dd, *J* = 7.8 Hz, 4.7 Hz, 1H), 7.64 (d, *J* = 8.8 Hz, 2H), 7.76 (d, *J* = 8.0 Hz, 1H), 7.79 (d, *J* = 8.8 Hz, 2H), 8.47 (dd, *J* = 4.7 Hz, 1.3 Hz, 1H), 8.57 (br s, 2H), 10.05 (s, 1H). ESI-MS (m/z): 474.71 [M+1].

1-(pyridin-3-ylmethyl)-3-(4-(pyrrolidin-1-ylsulfonyl)phenyl)thiourea (MS9). White solid (0.18 g, yield 48%). ^{1}H NMR (*d*-DMSO, 600 MHz) δ: 1.65-1.67 (m, 4H), 3.12-3.14 (m, 4H), 4.79 (d, *J* = 5.7 Hz, 2H), 7.37 (dd, *J* = 7.8 Hz, 4.8 Hz, 1H), 7.72 (d, *J* = 8.8 Hz, 2H), 7.76 (d, *J* = 7.8 Hz, 1H), 7.79 (d, *J* = 8.8 Hz, 2H), 8.47 (dd, *J* = 4.7 Hz, 1.2 Hz, 1H), 8.54 (s, 1H), 8.57 (d, *J* = 1.8 Hz, 1H), 10.01 (s, 1H). ESI-MS (m/z): 377.30 [M+1].

***N*-benzyl-4-(3-(pyridin-3-ylmethyl)thioureido)benzenesulfonamide (MS10).** White solid (0.19 g, yield 40%). ^{1}H NMR (*d*-DMSO, 600 MHz) δ: 3.97 (d, *J* = 6.3 Hz, 2H), 4.79 (d, *J* = 5.6 Hz, 2H), 7.22-7.25 (m, 3H), 7.28-7.30 (m, 2H), 7.38 (dd, *J* = 7.7 Hz, 4.6 Hz, 1H), 7.70 (d, *J* = 8.8 Hz, 2H), 7.74 (d, *J* = 8.8 Hz, 2H), 7.77 (d, *J* = 7.8 Hz, 1H), 8.04 (t, *J* = 6.3 Hz, 1H), 8.48 (dd, *J* = 4.6 Hz, 1.5 Hz, 1H), 8.54 (s, 1H), 8.58 (d, *J* = 1.8 Hz, 1H), 10.02 (s, 1H). ESI-MS (m/z): 413.54 [M+1].

1-(4-(morpholinosulfonyl)phenyl)-3-(pyridin-3-ylmethyl)thiourea (MS11). White solid (0.14 g, yield 35%). ^{1}H NMR (*d*-DMSO, 600 MHz) δ: 2.85 (t, *J* = 4.5 Hz, 4H), 3.63 (t, *J* = 4.5 Hz, 4H), 4.79 (d, *J* = 5.5 Hz, 2H), 7.38 (dd, *J* = 7.9 Hz, 4.8 Hz, 1H), 7.66 (d, *J* = 8.8 Hz, 2H), 7.77 (d, *J* = 7.8 Hz, 1H), 7.82 (d, *J* = 8.8 Hz, 2H), 8.47 (dd, *J* = 4.6 Hz, 1.3 Hz, 1H), 8.57 (d, *J* = 1.6 Hz, 1H), 8.61 (s, 1H), 10.12 (s, 1H). ESI-MS (m/z): 393.51 [M+1].

1-(4-((4-(4-fluorobenzyl)piperazin-1-yl)sulfonyl)phenyl)-3-(pyridin-3-ylmethyl)thiourea (MS22). White solid (0.056 g, yield 43%). ^{1}H NMR (*d*-DMSO, 600 MHz) δ: 2.38-2.45 (m, 4H), 2.85-2.92 (m, 4H), 3.44 (s, 2H), 4.80 (d, *J* = 5.5 Hz, 2H), 7.09 (t, *J* = 8.8 Hz, 2H), 7.26 (dd, *J* = 8.6 Hz, 6.0 Hz, 2H), 7.38 (dd, *J* = 7.9 Hz, 4.8 Hz, 1H), 7.64 (d, *J* = 8.8 Hz, 2H), 7.77 (d, *J* = 7.8 Hz, 1H), 7.79 (d, *J* = 8.8 Hz, 2H), 8.48 (dd, *J* = 4.7 Hz, 1.5 Hz, 1H), 8.57 (d, *J* = 1.5 Hz, 1H), 8.61 (s, 1H), 10.11 (s, 1H). ESI-MS (m/z): 500.55 [M+1].

1-(4-((4-(2,6-difluorobenzyl)piperazin-1-yl)sulfonyl)phenyl)-3-(pyridin-3-ylmethyl)thiourea (MS23). White solid (0.06 g, yield 48%). ^{1}H NMR (*d*-DMSO, 300 MHz) δ: 2.43-2.45 (m, 4H), 2.83-2.85 (m, 4H), 3.54 (s, 2H), 4.77 (d, *J* = 5.5 Hz, 2H), 7.05 (t, *J* = 7.9 Hz, 2H), 7.32-7.40 (m, 2H), 7.59 (d, *J* = 8.6 Hz, 2H), 7.74 (d, *J* = 8.6 Hz, 2H), 8.46 (d, *J* = 4.4 Hz, 1H), 8.55 (s, 1H), 8.61 (s, 1H), 10.08 (s, 1H). ESI-MS (m/z): 518.59 [M+1].

1-(4-((4-(3-fluorobenzyl)piperazin-1-yl)sulfonyl)phenyl)-3-(pyridin-3-ylmethyl)thiourea (MS24). White solid (0.062 g, yield 33%). ^{1}H NMR (*d*-DMSO, 600 MHz) δ: 2.43-2.45 (m, 4H), 2.89-2.92 (m, 4H), 3.48 (s, 2H), 4.80 (d, *J* = 5.4 Hz, 2H), 7.03-7.08 (m, 3H), 7.30-7.34 (m, 1H), 7.38 (dd, *J* = 7.7 Hz, 4.7 Hz, 1H), 7.64 (d, *J* = 8.8 Hz, 2H), 7.77 (dd, *J* = 7.7 Hz, 1.7 Hz, 1H), 7.79 (d, *J* = 8.8 Hz, 2H), 8.48 (dd, *J* = 4.7 Hz, 1.6 Hz, 1H), 8.57 (d, *J* = 2.0 Hz, 1H), 8.62 (br s, 1H), 10.12 (s, 1H). ESI-MS (m/z): 500.34 [M+1].

1-(4-((4-(2-fluorobenzyl)piperazin-1-yl)sulfonyl)phenyl)-3-(pyridin-3-ylmethyl)thiourea (MS25). White solid (0.082 g, yield 68%). ^{1}H NMR (*d*-DMSO, 600 MHz) δ: 2.42-2.48 (m, 4H), 2.88-2.92 (m, 4H), 3.51 (s, 2H), 4.79 (d, *J* = 5.2 Hz, 2H), 7.13 (t, *J* = 7.8 Hz, 2H), 7.27-7.33 (m, 2H), 7.38 (dd, *J* = 7.8 Hz, 4.8 Hz, 1H), 7.64 (d, *J* = 8.6 Hz, 2H), 7.77 (d, *J* = 8.1Hz, 1H), 7.80 (d, *J* = 8.6 Hz, 2H), 8.48 (d, *J* = 3.7 Hz, 1H), 8.57 (s, 1H), 8.63 (br s, 1H), 10.12 (s, 1H). ESI-MS (m/z): 500.63 [M+1].

1-(4-((4-(4-methylbenzyl)piperazin-1-yl)sulfonyl)phenyl)-3-(pyridin-3-ylmethyl)thiourea (MS26). White solid (0.074 g, yield 60%). ^{1}H NMR (*d*-DMSO, 300 MHz) δ: 2.23 (s, 3H), 2.24-2.42 (m, 4H), 2.79-2.89 (m, 4H), 3.38 (s, 2H), 4.79 (d, *J* = 5.3 Hz, 2H), 7.07-7.10 (m, 4H), 7.37 (dd, *J* = 7.8 Hz, 4.8 Hz, 1H), 7.61 (d, *J* = 8.6 Hz, 2H), 7.74-7.79 (m, 3H), 8.46 (d, *J* = 4.7 Hz, 1H), 8.56 (s, 1H), 8.62 (br s, 1H), 10.12 (s, 1H). ESI-MS (m/z): 496.37 [M+1].

1-(4-((4-(2-methylbenzyl)piperazin-1-yl)sulfonyl)phenyl)-3-(pyridin-3-ylmethyl)thiourea (MS27). White solid (0.07 g, yield 57%). ^{1}H NMR (*d*-DMSO, 300 MHz) δ: 2.21 (s, 3H), 2.39-2.44 (m, 4H), 2.81-2.87 (m, 4H), 3.40 (s, 2H), 4.77 (d, *J* = 5.4 Hz, 2H), 7.06-7.12 (m, 4H), 7.36 (dd, *J* = 7.7 Hz, 4.7 Hz, 1H), 7.62 (d, *J* = 8.7 Hz, 2H), 7.73-7.79 (m, 3H), 8.46 (d, *J* = 4.0 Hz, 1H), 8.56 (s, 1H), 8.61 (br s, 1H), 10.11 (s, 1H). ESI-MS (m/z): 496.68 [M+1].

1-(4-((4-(3-methylbenzyl)piperazin-1-yl)sulfonyl)phenyl)-3-(pyridin-3-ylmethyl)thiourea (MS28). White solid (0.063 g, yield 50%). ^{1}H NMR (*d*-DMSO, 300 MHz) δ: 2.23 (s, 3H), 2.34-2.43 (m, 4H), 2.82-2.89 (m, 4H), 3.39 (s, 2H), 4.78 (d, *J* = 5.6 Hz, 2H), 6.98-7.02 (m, 3H), 7.14 (t, *J* = 7.6 Hz, 1H), 7.37 (dd, *J* = 7.8 Hz, 4.8 Hz, 1H), 7.63 (d, *J* = 8.6 Hz, 2H), 7.74-7.78 (m, 3H), 8.46 (dd, *J* = 4.7 Hz, 1.6 Hz, 1H), 8.57 (d, *J* = 1.6 Hz, 1H), 8.64 (br s, 1H), 10.15 (s, 1H). ESI-MS (m/z): 496.54 [M+1].

1-(4-((4-(3-chlorobenzyl)piperazin-1-yl)sulfonyl)phenyl)-3-(pyridin-3-ylmethyl)thiourea (MS29). White solid (0.075 g, yield 58%). ^{1}H NMR (*d*-DMSO, 300 MHz) δ: 2.38-2.43 (m, 4H), 2.83-2.90 (m, 4H), 3.45 (s, 2H), 4.78 (d, *J* = 5.4 Hz, 2H), 7.18 (t, *J* = 6.3 Hz, 1H), 7.27-7.30 (m, 3H), 7.37 (dd, *J* = 7.8 Hz, 4.7 Hz, 1H), 7.63 (d, *J* = 8.7 Hz, 2H), 7.74-7.80 (m, 3H), 8.46 (dd, *J* = 4.7 Hz, 1.3 Hz, 1H), 8.56 (d, *J* = 1.5 Hz, 1H), 8.62 (br s, 1H), 10.11 (s, 1H). ESI-MS (m/z): 517.10 [M+1].

1-(4-((4-(2-chlorobenzyl)piperazin-1-yl)sulfonyl)phenyl)-3-(pyridin-3-ylmethyl)thiourea (MS30). White solid (0.04 g, yield 31%). ^{1}H NMR (*d*-DMSO, 300 MHz) δ: 2.43-2.49 (m, 4H), 2.82-2.92 (m, 4H), 3.55 (s, 2H), 4.78 (d, *J* = 5.4 Hz, 2H), 7.23-7.28 (m, 2H), 7.34-7.39 (m, 3H), 7.63 (d, *J* = 8.7 Hz, 2H), 7.74-7.80 (m, 3H), 8.46 (d, *J* = 4.5 Hz, 1H), 8.56 (d, *J* = 1.7 Hz, 1H), 8.63 (br s, 1H), 10.13 (s, 1H). ESI-MS (m/z): 517.13 [M+1].

1-(4-((4-(4-chlorobenzyl)piperazin-1-yl)sulfonyl)phenyl)-3-(pyridin-3-ylmethyl)thiourea (MS31). White solid (0.06 g, yield 45%). ^{1}H NMR (*d*-DMSO, 300 MHz) δ: 2.37-2.43 (m, 4H), 2.82-2.89 (m, 4H), 3.43 (s, 2H), 4.78 (d, *J* = 5.4 Hz, 2H), 7.23 (d, *J* = 8.4 Hz, 2H), 7.32 (d, *J* = 8.4 Hz, 2H), 7.37 (dd, *J* = 7.7 Hz, 4.8 Hz, 1H), 7.63 (d, *J* = 8.7 Hz, 2H), 7.74-7.80 (m, 3H), 8.46 (dd, *J* = 4.7 Hz, 1.2 Hz, 1H), 8.56 (d, *J* = 1.5 Hz, 1H), 8.64 (br s, 1H), 10.14 (s, 1H). ESI-MS (m/z): 517.11 [M+1].

1-(4-((4-(4-bromobenzyl)piperazin-1-yl)sulfonyl)phenyl)-3-(pyridin-3-ylmethyl)thiourea

(MS32). White solid (0.067 g, yield 47%). ^{1}H NMR (*d*-DMSO, 300 MHz) δ: 2.38-2.46 (m, 4H), 2.82-2.89 (m, 4H), 3.47 (s, 2H), 4.78 (d, *J* = 5.5 Hz, 2H), 7.24 (d, *J* = 8.1 Hz, 2H), 7.35 (d, *J* = 8.1 Hz, 2H), 7.38 (d, *J* = 5.4 Hz, 1H), 7.63 (d, *J* = 8.6 Hz, 2H), 7.74-7.80 (m, 3H), 8.47 (dd, *J* = 4.6 Hz, 1.3 Hz, 1H), 8.56 (s, 1H), 8.63 (br s, 1H), 10.13 (s, 1H). ESI-MS (m/z): 566.71 [M+1].

1-(4-((4-(naphthalen-2-ylmethyl)piperazin-1-yl)sulfonyl)phenyl)-3-(pyridin-3-ylmethyl)thiourea (MS33). White solid (0.063 g, yield 46%). ^{1}H NMR (*d*-DMSO, 300 MHz) δ: 2.84-2.93 (m, 4H), 3.04-3.11 (m, 4H), 3.62 (s, 2H), 4.78 (d, *J* = 5.4 Hz, 2H), 7.34-7.40 (m, 2H), 7.44-7.47 (m, 2H), 7.62 (d, *J* = 8.7 Hz, 2H), 7.72-7.86 (m, 7H), 8.47 (d, *J* = 4.1 Hz, 1H), 8.56 (s, 1H), 8.66 (br s, 1H), 10.17 (s, 1H). ESI-MS (m/z): 532.77 [M+1].

1-(pyridin-3-ylmethyl)-3-(4-((4-(4-(trifluoromethyl)benzyl)piperazin-1-yl)sulfonyl)phenyl)thiourea (MS34). White solid (0.07 g, yield 51%). ^{1}H NMR (*d*-DMSO, 300 MHz) δ: 2.41-2.46 (m, 4H), 2.81-2.91 (m, 4H), 3.54 (s, 2H), 4.78 (d, *J* = 5.5 Hz, 2H), 7.18 (d, *J* = 8.2 Hz, 2H), 7.36 (dd, *J* = 7.6 Hz, 4.7 Hz, 1H), 7.45 (d, *J* = 7.8 Hz, 2H), 7.61-7.64 (m, 4H), 7.74-7.80 (m, 3H), 8.47 (d, *J* = 3.5 Hz, 1H), 8.56 (d, *J* = 1.5 Hz, 1H), 8.62 (br s, 1H), 10.11 (s, 1H). ESI-HRMS (m/z): 550.1564 [M+1].

1-(pyridin-3-ylmethyl)-3-(4-((4-(4-(trifluoromethoxy)benzyl)piperazin-1-yl)sulfonyl)phenyl)thiourea (MS35). White solid (0.067 g, yield 47%). ^{1}H NMR (*d*-DMSO, 300 MHz) δ: 2.38-2.46 (m, 4H), 2.82-2.89 (m, 4H), 3.47 (s, 2H), 4.78 (d, *J* = 5.5 Hz, 2H), 7.24 (d, *J* = 8.1 Hz, 2H), 7.35 (d, *J* = 8.1 Hz, 2H), 7.38 (d, *J* = 5.4 Hz, 1H), 7.63 (d, *J* = 8.6 Hz, 2H), 7.74-7.80 (m, 3H), 8.47 (dd, *J* = 4.6 Hz, 1.3 Hz, 1H), 8.56 (s, 1H), 8.63 (br s, 1H), 10.13 (s, 1H). ESI-MS (m/z): 566.71 [M+1].

1-(4-((4-(4-butylbenzyl)piperazin-1-yl)sulfonyl)phenyl)-3-(pyridin-3-ylmethyl)thiourea (MS36). White solid (0.07 g, yield 52%). ^{1}H NMR (*d*-DMSO, 300 MHz) δ: 0.86 (t, *J* = 7.4 Hz, 3H), 1.21-1.29 (m, 4H), 1.44-1.52 (m, 2H), 2.35-2.42 (m, 4H), 2.82-2.89 (m, 4H), 3.39 (s, 2H), 4.78 (d, *J* = 5.5 Hz, 2H), 7.05-7.12 (m, 4H), 7.34-7.38 (m, 1H), 7.62 (d, *J* = 8.7 Hz, 2H), 7.74-7.80 (m, 3H), 8.46 (d, *J* = 4.3 Hz, 1H), 8.56 (s, 1H), 8.65 (br s, 1H), 10.17 (s, 1H). ESI-MS (m/z): 538.66 [M+1].

1-(4-((4-(4-nitrobenzyl)piperazin-1-yl)sulfonyl)phenyl)-3-(pyridin-3-ylmethyl)thiourea (MS37). White solid (0.12 g, yield 41%). ^{1}H NMR (*d*-DMSO, 300 MHz) δ: 2.42-2.46 (m, 4H), 2.85-2. 92 (m, 4H), 3.61 (s, 2H), 4.78 (d, *J* = 5.4 Hz, 2H), 7.37 (dd, *J* = 7.8 Hz, 4.7 Hz, 1H), 7.52 (d, *J* = 8.7 Hz, 2H), 7.63 (d, *J* = 8.7 Hz, 2H), 7.76 (d, *J* = 7.8 Hz, 1H), 7.82 (d, *J* = 8.7 Hz, 2H), 8.13 (d, *J* = 8.7 Hz, 2H), 8.47 (dd, *J* = 4.7 Hz, 1.4 Hz, 1H), 8.56 (d, *J* = 2.1 Hz, 1H), 8.68 (br s, 1H), 10.21 (s, 1H). ESI-MS (m/z): 527.70 [M+1].

1-(4-((4-(4-aminobenzyl)piperazin-1-yl)sulfonyl)phenyl)-3-(pyridin-3-ylmethyl)thiourea (MS38).10% Pd/C (0.01 g) was added to a stirring solution of **MS37** (50mg, 0.1mmol) in EtOAc (10 mL), and the reaction mixture was stirred under hydrogen atmosphere for 12 h at room temperature. The mixture was diluted with water (50 mL) and then extracted with EtOAc (3× 50 mL). The combinedorganic layers were washed with saturated sodium chloride solution (3 ×50mL), dried over anhydrous Na_2SO_4 and concentratedunder reduced pressure. The residue was purified by silica gel column chromatography (gradient CH_2Cl_2 : MeOH = 100:1 to 100:3) to give compound**MS38** as a white solid (34 mg, yield 72%). ^{1}H NMR (*d*-DMSO, 600 MHz) *δ*: 2.43-2.48 (m, 4H), 2.82-2. 90 (m, 4H), 3.71 (s, 2H), 4.79 (d, *J* = 5.5 Hz, 2H), 5.09 (s, 2H), 7.36 (dd, *J* = 7.8 Hz, 4.7 Hz, 1H), 7.52 (d, *J* = 8.8 Hz, 2H), 7.63 (d, *J* = 8.8 Hz, 2H), 7.77 (d, *J* = 7.8 Hz, 1H), 7.88 (d, *J* = 8.6 Hz, 2H), 8.13 (d, *J* = 8.6 Hz, 2H), 8.47 (dd, *J* = 4.7 Hz, 1.4 Hz, 1H), 8.56 (s, 1H), 8.68 (br s, 1H), 10.19 (s, 1H). ESI-MS (m/z): 497.71 [M+1].

1-(4-((4-((1*H*-benzo[d]imidazol-2-yl)methyl)piperazin-1-yl)sulfonyl)phenyl)-3-(pyridin-3-ylmethyl)thiourea (MS39).White solid (0.098 g, yield 76%). ^{1}H NMR (*d*-DMSO, 600 MHz) *δ*: 2.54-2.55 (m, 4H), 2.92-2.94 (m, 4H), 3.73 (s, 2H), 4.79 (d, *J* = 6.0 Hz, 2H), 7.09-7.12 (m, 2H), 7.37-7.39 (m, 1H), 7.41-7.46 (m, 2H), 7.66 (d, *J* = 8.9 Hz, 2H), 7.77 (dd, *J* = 7.8 Hz, 1.7 Hz, 2H), 7.81 (d, *J* = 8.9 Hz, 2H), 8.47 (dd, *J* = 4.7 Hz, 1.6 Hz, 1H), 8.57 (d, *J* = 1.7 Hz, 1H), 8.62 (br s, 1H), 10.13 (s, 1H), 12.17 (s, 1H). ESI-MS (m/z): 522.63 [M+1].

1-(4-((4-(furan-3-ylmethyl)piperazin-1-yl)sulfonyl)phenyl)-3-(pyridin-3-ylmethyl)thiourea (MS40). White solid (0.065 g, yield 55%). ^{1}H NMR (*d*-DMSO, 600 MHz) *δ*: 2.52-2.61 (m, 4H), 2.85-2.94 (m, 4H), 3.39 (s, 2H), 4.77 (d, *J* = 5.5 Hz, 2H), 6.38 (s, 1H), 7.36 (dd, *J* = 7.8 Hz, 4.6 Hz, 1H), 7.57-7.63 (m, 4H), 7.74 (d, *J* = 7.8 Hz, 1H), 7.85 (d, *J* = 8.7 Hz, 2H), 8.44 (d, *J* = 4.6 Hz, 1H), 8.54 (s, 1H), 8.83 (t, *J* = 5.5 Hz, 1H), 10.44 (s, 1H). ESI-MS (m/z): 472.62 [M+1].

1-(pyridin-3-ylmethyl)-3-(4-((4-(thiophen-3-ylmethyl)piperazin-1-yl)sulfonyl)phenyl)thiourea (MS41). White solid (0.06 g, yield 49%). ^{1}H NMR (*d*-DMSO, 600 MHz) *δ*: 2.55-2.62 (m, 4H), 2.83-2.93 (m, 4H), 3.41 (s, 2H), 4.77 (d, *J* = 5.5 Hz, 2H), 6.96 (d, *J* = 4.4 Hz, 1H), 7.36 (dd, *J* = 7.7 Hz, 4.6 Hz, 2H), 7.45 (s, 1H), 7.62 (d, *J* = 8.5 Hz, 2H), 7.74 (d, *J* = 7.7 Hz, 1H), 7.80 (d, *J* = 8.5 Hz, 2H), 8.44 (dd, *J* = 4.9 Hz, 1.6 Hz, 1H), 8.55 (d, *J* = 2.2 Hz, 1H), 8.72 (s, 1H), 10.27 (s, 1H). ESI-MS (m/z): 488.77 [M+1].

1-(4-((4-(furan-2-ylmethyl)piperazin-1-yl)sulfonyl)phenyl)-3-(pyridin-3-ylmethyl)thiourea (MS42). White solid (0.07 g, yield 59%). ^{1}H NMR (*d*-DMSO, 600 MHz) *δ*: 2.47-2.52 (m, 4H), 2.83-2.88 (m, 4H), 3.68 (s, 2H), 4.78 (d, *J* = 5.5 Hz, 2H), 6.89-6.92 (m, 2H), 7.33-7.39

(m, 2H), 7.62 (d, *J* = 8.7 Hz, 2H), 7.75 (d, *J* = 7.9 Hz, 1H), 7.86 (d, *J* = 8.7 Hz, 2H), 8.45 (d, *J* = 4.7 Hz, 1H), 8.55 (d, *J* = 1.6 Hz, 1H), 8.86 (t, *J* = 5.8 Hz, 1H), 10.46 (s, 1H). ESI-MS (m/z): 472.55 [M+1].

1-(pyridin-3-ylmethyl)-3-(4-((4-(thiophen-2-ylmethyl)piperazin-1-yl)sulfonyl)phenyl)thiourea (MS43). White solid (0.065 g, yield 56%). ^{1}H NMR (*d*-DMSO, 600 MHz) δ: 2.43-2.47 (m, 4H), 2.83-2.89 (m, 4H), 3.56 (s, 2H), 4.78 (d, *J* = 5.5 Hz, 2H), 6.27 (d, *J* = 3.0 Hz, 1H), 6.35-6.37 (m, 1H), 7.35 (dd, *J* = 7.7 Hz, 4.9 Hz, 1H), 7.55 (s, 1H), 7.62 (d, *J* = 8.7 Hz, 2H), 7.75 (d, *J* = 7.7 Hz, 1H), 7.82 (d, *J* = 8.7 Hz, 2H), 8.45 (d, *J* = 3.5 Hz, 1H), 8.55 (s, 1H), 8.77 (t, *J* = 5.5 Hz, 1H), 10.32 (s, 1H). ESI-MS (m/z): 488.79 [M+1].

1-(pyridin-3-ylmethyl)-3-(4-((4-(pyridin-4-ylmethyl)piperazin-1-yl)sulfonyl)phenyl)thiourea (MS44). White solid (0.06 g, yield 52%). ^{1}H NMR (*d*-DMSO, 600 MHz) δ: 2.44-2.47 (m, 4H), 2.88-2. 92 (m, 4H), 3.52 (s, 2H), 4.79 (d, *J* = 5.7 Hz, 2H), 7.26 (d, *J* = 5.9 Hz, 2H), 7.37-7.39 (m, 1H), 7.65 (d, *J* = 8.8 Hz, 2H), 7.76-7.78 (m, 1H), 7.81 (d, *J* = 8.8 Hz, 2H), 8.46 (dd, *J* = 4.5 Hz, 1.3 Hz, 2H), 8.47 (d, *J* = 4.7 Hz, 1.5 Hz, 1H), 8.57 (d, *J* = 1.8 Hz, 1H), 8.63 (br s, 1H), 10.15 (s, 1H). ESI-MS (m/z): 483.37 [M+1].

1-(pyridin-3-ylmethyl)-3-(4-((4-(pyridin-3-ylmethyl)piperazin-1-yl)sulfonyl)phenyl)thiourea (MS45). White solid (0.065 g, yield 57%). ^{1}H NMR (*d*-DMSO, 600 MHz) δ: 2.45-2.48 (m, 4H), 2.87-2. 92 (m, 4H), 3.54 (s, 2H), 4.79 (d, *J* = 5.7 Hz, 2H), 7.31 (dd, *J* = 7.8 Hz, 4.8 Hz, 1H), 7.38 (dd, *J* = 7.8 Hz, 4.8 Hz, 1H), 7.62-7.65 (m, 3H), 7.75-7.77 (m, 1H), 7.79-7.80 (m, 2H), 8.43 (d, *J* = 1.6 Hz, 1H), 8.44 (dd, *J* = 4.7 Hz, 1.4 Hz, 1H), 8.47 (dd, *J* = 4.7 Hz, 1.4 Hz, 1H), 8.57 (d, *J* = 1.8 Hz, 1H), 8.64 (br s, 1H), 10.15 (s, 1H). ESI-MS (m/z): 483.38 [M+1].

1-(pyridin-3-ylmethyl)-3-(4-((4-(p-tolyl)piperazin-1-yl)sulfonyl)phenyl)thiourea (MS46). White solid (0.25 g, yield 51%). ^{1}H NMR (*d*-DMSO, 600 MHz) δ: 2.17 (s, 3H), 2.98 (t, *J* = 4.8 Hz, 4H), 3.12 (t, *J* = 4.8 Hz, 4H), 4.78 (d, *J* = 5.4 Hz, 2H), 6.79 (d, *J* = 8.4 Hz, 2H), 7.00 (d, *J* = 8.4 Hz, 2H), 7.35 (dd, *J* = 7.8 Hz, 4.8 Hz, 1H), 7.68 (d, *J* = 9.0 Hz, 2H), 7.75 (d, *J* = 7.8 Hz, 1H), 7.80 (d, *J* = 9.0 Hz, 2H), 8.44 (dd, *J* = 4.8 Hz, 1.8 Hz, 1H), 8.55 (d, *J* = 1.2 Hz, 1H), 8.60 (br s, 1H), 10.10 (s, 1H). ESI-MS (m/z): 482.66 [M+1].

Scheme 2

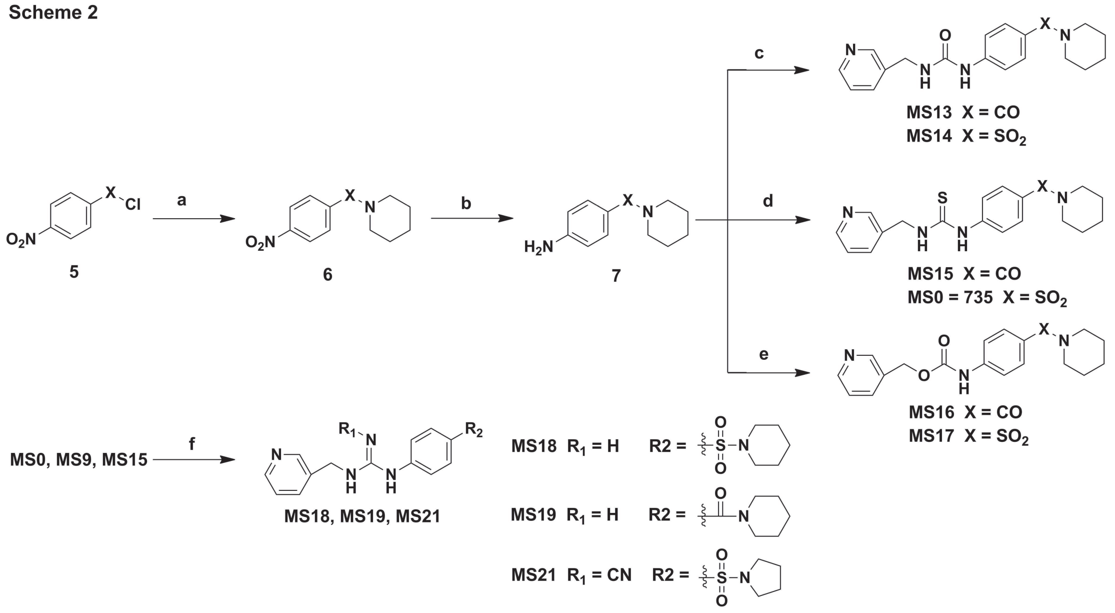

Reagents and conditions: (a) piperidine, CH_2Cl_2, RT, 2 h, yield 100%; (b)Pd/C, H_2, EtOAc, RT, 12 h, yield 70-74%; (c) triphosgene, THF, 50℃, 2 h, yield 39-44%; (d) thiophosgene, $NaHCO_3$, CH_2Cl_2, C_2H_5OH,RT, 1 h, yield 47-75%;(e) pyridin-3-ylmethyl carbonochloridate, THF, RT, 3 h, yield 58-60%; (f) $NH_3{\cdot}OH$, $NaIO_4$, DMF, 70℃, 6 h (or cyanamide, EDC, Et_3N, THF, 50℃, 12h), yield 40-71%;

Experimental section

1-(4-(piperidine-1-carbonyl)phenyl)-3-(pyridin-3-ylmethyl)urea (MS13). To a stirred solution of (4-aminophenyl)(piperidin-1-yl)methanone[18] (**7a**, 0.2 g, 1 mmol) in THF (20 mL), triphosgene (0.12 g, 0.4 mmol) was added in one portion and stirred for 2 h at 50℃. The reaction mixture was cooled to 0 ℃, pyridin-3-ylmethanamine (0.11 g, 1 mmol) was introduced and continuously stirred at room temperature for 1 h. The solvent was concentrated in vacuo and the crude product was purified by column chromatography (CH_2Cl_2 : MeOH = 100:2) to give compound **MS13** as a white solid (0.13 g, yield 39%). ^1H NMR (*d*-DMSO, 600 MHz) δ: 1.44-1.49 (m, 4H), 1.58-1.62 (m, 2H), 3.40-3.44 (m, 4H), 4.33 (d, J = 5.4 Hz, 2H), 6.77 (t, J = 6.0 Hz, 1H), 7.25 (d, J = 8.4 Hz, 2H), 7.36 (dd, J = 7.8 Hz, 4.8 Hz, 1H), 7.44 (d, J = 8.4 Hz, 2H), 7.71 (d, J = 7.8 Hz, 1H), 8.45 (dd, J = 7.8 Hz, 1.2 Hz, 1H), 8.52 (s, 1H), 8.82 (s, 1H). ESI-MS (m/z): 339.61 [M+1].

The synthetic method for target compounds **MS14** was similar to the synthesis of compound **MS13**.

1-(4-(piperidin-1-ylsulfonyl)phenyl)-3-(pyridin-3-ylmethyl)urea (MS14). White solid (0.16 g, yield 44%). ^1H NMR (*d*-DMSO, 600 MHz) δ: 1.34-1.35 (m, 2H), 1.50-1.54 (m, 4H),

2.81-2.84 (m, 4H), 4.34 (d, *J* = 6.0 Hz, 2H), 6.90 (t, *J* = 5.4 Hz, 1H), 7.34-7.37 (m, 1H), 7.56 (d, *J* = 8.4 Hz, 2H), 7.62 (d, *J* = 8.4 Hz, 2H), 7.70-7.72 (m, 1H), 8.45 (dd, *J* = 4.8 Hz, 1.8 Hz, 1H), 8.53 (s, 1H), 9.16 (s, 1H). ESI-MS (m/z): 335.55 [M+1].

1-(4-(piperidin-1-ylsulfonyl)phenyl)-3-(pyridin-3-ylmethyl)thiourea (MS0). White solid (0.12 g, 75%). ^{1}H NMR (*d*-DMSO, 600 MHz) δ: 1.37-1.42 (m, 2H), 1.52-1.60 (m, 4H), 2.86-2.88 (m, 4H), 4.78 (d, *J* = 5.4 Hz, 2H), 7.37 (dd, *J* = 7.8 Hz, 4.8 Hz, 1H), 7.65 (d, *J* = 7.2 Hz,, 2H), 7.75 (d, *J* = 1.8 Hz, 2H), 7.78 (d, *J* = 7.2 Hz, 2H),8.47 (dd, *J* = 4.8 Hz, 1.2 Hz, 1H), 8.57 (d, *J* = 1.7 Hz, 1H), 8.60 (s, 1H), 10.08 (s, 1H). ESI-MS (m/z): 391.18 [M+1].

1-(4-(piperidine-1-carbonyl)phenyl)-3-(pyridin-3-ylmethyl)thiourea (MS15). White solid (0.16 g, 47%). ^{1}H NMR (*d*-DMSO, 600 MHz) δ: 1.49-1.53 (m, 4H), 1.60-1.63 (m, 2H), 3.41-3.47 (m, 4H), 4.78 (d, *J* = 5.4 Hz, 2H), 7.33 (d, *J* = 8.4 Hz, 2H), 7.37 (d, *J* = 5.4 Hz,, 1H), 7.50 (d, *J* = 8.4 Hz, 2H), 8.34 (s, 1H), 8.46 (dd, *J* = 4.2 Hz, 1.2 Hz, 1H), 8.56 (s, 1H), 9.76 (s, 1H). ESI-MS (m/z): 355.50 [M+1].

pyridin-3-ylmethyl (4-(piperidine-1-carbonyl)phenyl)carbamate (MS16).A solution of (4-aminophenyl)(piperidin-1-yl)methanone (**7a**,0.2 g, 1mmol) and pyridin-3-ylmethyl carbonochloridate (0.21 g, 1.2mmol) in THF (20 mL) was stirred atroom temperature for 3 h. Then solvent was concentrated in vacuo and the crude product was purified by column chromatography(gradient CH_2Cl_2 : MeOH =100:1 to 100:2) to give compound **MS16** as a white solid (0.19 g, yield 58%). ^{1}H NMR (*d*-DMSO, 600 MHz) δ: 1.45-1.49 (m, 4H), 1.60-1.62 (m, 2H), 3.40-3.45 (m, 4H), 5.21 (s, 2H), 7.30 (d, *J* = 8.4 Hz, 2H), 7.42-7.44 (m, 1H), 7.51 (d, *J* = 8.4 Hz, 2H), 7.84-7.86 (m, 1H), 8.55 (dd, *J* = 4.8 Hz, 1.8 Hz, 1H), 8.66 (s, 1H), 9.31 (s, 1H). ESI-MS (m/z): 338.61 [M-1].

The synthetic method for target compounds **MS17** was similar to the synthesis of compound **MS16**

pyridin-3-ylmethyl (4-(piperidin-1-ylsulfonyl)phenyl)carbamate (MS17). White solid (0.22 g, yield 60%). ^{1}H NMR (*d*-DMSO, 600 MHz) δ: 1.34-1.35 (m, 2H), 1.50-1.54 (m, 4H), 2.82-2.84 (m, 4H), 5.23 (s, 2H), 7.44 (dd, *J* = 8.4 Hz, 4.8 Hz, 1H), 7.65 (d, *J* = 8.4 Hz, 2H), 7.68 (d, *J* = 8.4 Hz, 2H), 7.87 (d, *J* = 7.8 Hz, 1H), 8.56 (s, 1H), 8.67 (d, *J* = 1.8 Hz, 1H), 10.29 (s, 1H). ESI-MS (m/z): 376.54 [M+1].

1-(4-(piperidin-1-ylsulfonyl)phenyl)-3-(pyridin-3-ylmethyl)guanidine (MS18). To a

stirred solution of **735** (30mg, 0.077mmol) and $NaIO_4$ (20 mg, 0.09 mmol) in DMF (3 mL), ammonia solution (3 mL) was added and stirred for 6h at 70℃. The mixture was diluted with water (10 mL) and then extracted with CH_2Cl_2 (3×20 mL). The combined organic layers were washed with saturated sodium chloride solution (3 ×20mL), dried over anhydrous Na_2SO_4 and concentrated under reduced pressure. The residue was purified by silica gel column chromatography (gradient CH_2Cl_2 : MeOH = 100:1, 100:2) to give compound **MS18** as a white solid (20 mg, yield 71%). ^{1}H NMR (*d*-DMSO, 300 MHz) δ: 1.22-1.24 (m, 2H), 1.32-1.34 (m, 4H), 2.79-2.82 (m, 4H), 4.38 (s, 2H), 5.54 (br s, 2H), 6.36 (s, 1H), 6.92 (d, *J* = 6.6 Hz, 2H), 7.36 (dd, *J* = 7.8 Hz, 4.8 Hz, 1H), 7.47 (d, *J* = 8.7 Hz, 2H), 7.74 (d, *J* = 7.8 Hz, 1H), 8.44 (dd, *J* = 4.5 Hz, 1.5 Hz, 1H), 8.53 (d, *J* = 1.5 Hz, 1H). ESI-MS (m/z): 374.54 [M+1].

The synthetic method for target compounds **MS19** was similar to the synthesis of compound **MS18**

1-(4-(piperidine-1-carbonyl)phenyl)-3-(pyridin-3-ylmethyl)guanidine (MS19). White solid (18 mg, yield 64%). ^{1}H NMR (*d*-DMSO, 300 MHz) δ: 1.29-1.34 (m, 2H), 1.52-1.54 (m, 4H), 2.79-2.83 (m, 4H), 4.42 (s, 2H), 5.55 (br s, 2H), 6.36 (s, 1H), 7.03 (d, *J* = 7.8 Hz, 2H), 7.35-7.39 (m, 1H), 7.51 (d, *J* = 7.8 Hz, 2H), 7.74 (d, *J* = 8.1 Hz, 1H), 8.45-8.46 (m, 1H), 8.55 (s, 1H). ESI-MS (m/z): 338.44 [M+1].

2-Cyano-1-(pyridin-3-ylmethyl)-3-(4-(pyrrolidin-1-ylsulfonyl)phenyl)guanidine (MS21). A solution of compound **MS9** (0.1 g, 0.26mmol), EDC (0.1 g, 0.55mmol), Et_3N (0.37 mL) and cyanamide (0.22 g, 5.2 mmol) in THF (20 mL) was stirred at 50℃ for 12 h. Then, the solvent was removed under reduced pressure and the residue was purified by silica gel column chromatography (gradient CH_2Cl_2 : MeOH = 100:1 to 100:10)to afford compound **MS21** as a white solid (0.04g, yield 40%). ^{1}H NMR (*d*-DMSO, 600 MHz) δ: 1.64-1.66 (m, 4H), 3.11-3.13 (m, 4H), 4.50 (d, *J* = 5.8 Hz, 2H), 7.39 (dd, *J* = 7.8 Hz, 4.7 Hz, 1H), 7.45 (d, *J* = 8.6 Hz, 2H), 7.73-7.75 (m, 3H), 8.20 (t, *J* = 5.9 Hz, 1H), 8.48 (dd, *J* = 4.6 Hz, 1.5 Hz, 1H), 8.54 (d, *J* = 1.6 Hz, 1H), 9.56 (s, 1H). ESI-MS (m/z): 385.49 [M+1].

Scheme 3

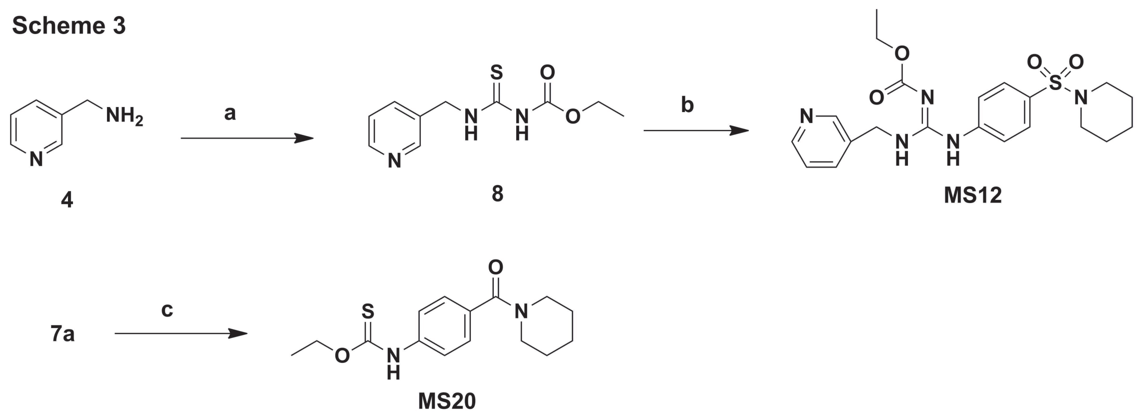

Reagents and conditions: (a)ethoxycarbonylisothiocyanate, C_2H_5OH, RT, 1 h, yield 94%; (b) 4-(piperidin-1-ylsulfonyl)aniline, EDC, Et_3N, CH_2Cl_2, 30℃,38 h, yield 20%; (c) C_2H_5OH, refluxed, 6 h, yield 55%;

Experimental section

1-(4-(ethoxycarbonyl))-3-(pyridin-3-ylmethyl)thiourea (8). A solution of compound **4** (0.98 g, 9.2mmol) and ethoxycarbonylisothiocyanate (1 g, 7.6mmol) in C_2H_5OH (20 mL) was stirred at room temperature for 1 h. Then, the solvent was removed under reduced pressure and the residue was purified by silica gel column chromatography (CH_2Cl_2 : MeOH = 100:1) to afford compound **8** as a white solid (1.7 g, yield 94%). ^{1}H NMR (*d*-DMSO, 600 MHz) δ: 1.22 (t, J = 7.2 Hz, 3H), 4.16 (m, 2H), 4.84 (d, J = 6.0 Hz,2H), 7.35 (dd, J = 8.4 Hz, 4.8 Hz,1H), 7.74 (d, J = 7.8 Hz,1H), 8.46 (dd, J = 4.8 Hz, 1.8 Hz,1H),8.55 (d, J = 1.8 Hz,1H),10.30 (s, 1H), 11.06 (s, 1H).

2-(4-(ethoxycarbonyl))-1-(4-(piperidin-1-ylsulfonyl)phenyl)-3-(pyridin-3-ylmethyl)guanidine (MS12). A solution of compound **8** (0.28 g, 1.2 mmol), EDC (0.48 g, 2.4 mmol), Et_3N (1.7 mL) and 4-(piperidin-1-ylsulfonyl)aniline (0.3 g, 1.2 mmol) in CH_2Cl_2 (20 mL) was stirred at 30℃ for 38 h. Then, the solvent was removed under reduced pressure and the residue was purified by silica gel column chromatography (CH_2Cl_2 : MeOH = 100:3) to give compound **MS12** as a white solid (0.1 g, yield 20%). ^{1}H NMR (*d*-DMSO, 600 MHz) δ: 1.13 (t, J = 6.6 Hz, 3H), 1.33-1.37 (m, 2H), 1.51-1.56 (m, 4H), 2.82-2.87 (m, 4H), 3.95-3.97 (m, 2H), 4.61 (s, 2H), 7.36-7.39 (m, 3H), 7.57-66 (m, 3H), 7.76 (d, J = 5.5 Hz, 1H), 8.44-8.48 (m, 1H), 8.57 (s, 1H), 10.02 (s, 1H). ESI-MS (m/z): 446.54 [M+1].

***O*-ethyl (4-(piperidine-1-carbonyl)phenyl)carbamothioate (MS20).** A solution of compound (4-aminophenyl)(piperidin-1-yl)methanone(**7a**, 0.27 g, 1.3 mmol) in C_2H_5OH (20 mL) was stirred refluxed for 6 h. Then, the solvent was removed under reduced pressure and

the residue was purified by silica gel column chromatography(CH_2Cl_2 : MeOH = 100:2)to give compound **MS20** as a white solid (0.21 g, yield 55%). ^{1}H NMR ($CDCl_3$, 600 MHz) δ: 1.15 (t, J = 6.0 Hz, 3H), 1.33-1.37 (m, 2H), 1.51-1.56 (m, 4H), 2.82-2.87 (m, 4H), 3.95-3.97 (m, 2H), 6.46 (d, J = 8.4 Hz, 2H), 6.82 (d, J = 8.4 Hz, 2H). ESI-MS (m/z): 293.41 [M+1].

Supplemental References

1. Bedwell, D.M., Strobel, S.A., Yun, K., Jongeward, G.D. & Emr, S.D. Sequence and structural requirements of a mitochondrial protein import signal defined by saturation cassette mutagenesis. *Mol Cell Biol* **9**, 1014-1025 (1989).
2. Lv, X.Q., Zhang, R.Y., Xu, X.W., Guan, Y.F. & Miao, C.Y. Expression, purification, and enzymatic activity assay of nicotinamide mononucleotide adenylyltransferase. *Acad J Sec Mil Med Univ* **31**, 1251-1254 (2010).
3. Zhang, R.Y. *et al.* A fluorometric assay for high-throughput screening targeting nicotinamide phosphoribosyltransferase. *Anal Biochem* **412**, 18-25 (2011).
4. Rowlands, M.G. *et al.* High-throughput screening assay for inhibitors of heat-shock protein 90 ATPase activity. *Anal Biochem* **327**, 176-183 (2004).
5. Zhang, J.H., Chung, T.D. & Oldenburg, K.R. A Simple Statistical Parameter for Use in Evaluation and Validation of High Throughput Screening Assays. *J Biomol Screen* **4**, 67-73 (1999).
6. Matsumura, H. & Miyachi, S. Cycling Assay for Nicotinamide Adenine Dinucleotides. *Methods In Enzymology* **69**, 465-470 (1980).
7. Wang, P. *et al.* Loss of AMP-activated protein kinase-alpha2 impairs the insulin-sensitizing effect of calorie restriction in skeletal muscle. *Diabetes* **61**, 1051-1061 (2012).
8. Wang, P. *et al.* Nicotinamide phosphoribosyltransferase protects against ischemic stroke through SIRT1-dependent adenosine monophosphate-activated kinase pathway. *Ann Neurol* **69**, 360-374 (2011).
9. Wang, P. *et al.* Perivascular adipose tissue-derived visfatin is a vascular smooth muscle cell growth factor: role of nicotinamide mononucleotide. *Cardiovasc Res* **81**, 370-380 (2009).
10. Zhu, H. *et al.* R16, a novel amonafide analogue, induces apoptosis and G2-M arrest via poisoning topoisomerase II. *Mol Cancer Ther* **6**, 484-495 (2007).
11. Cheng, X.L. *et al.* Methotrexate and 5-aminoimidazole-4-carboxamide riboside exert synergistic anticancer action against human breast cancer and hepatocellular carcinoma. *Acta Pharmacol Sin* **34**, 951-959 (2013).
12. Liu, S. *et al.* Enhancement of cytotoxicity of antimicrobial peptide magainin II in tumor cells by bombesin-targeted delivery. *Acta Pharmacol Sin* **32**, 79-88 (2011).
13. Xu, T.Y. *et al.* Chronic exposure to nicotine enhances insulin sensitivity through alpha7 nicotinic acetylcholine receptor-STAT3 pathway. *PLoS One* **7**, e51217 (2012).
14. Scheuermann, T.H. *et al.* Allosteric inhibition of hypoxia inducible factor-2 with small molecules. *Nat Chem Biol* **9**, 271-276 (2013).
15. Martinez Molina, D. *et al.* Monitoring drug target engagement in cells and tissues using the cellular thermal shift assay. *Science* **341**, 84-87 (2013).
16. Wang, P. *et al.* Vascular smooth muscle cell apoptosis is an early trigger for hypothyroid atherosclerosis. *Cardiovasc Res* (2014).
17. Sanner, M.F. Python: a programming language for software integration and

development. *J Mol Graph Model* **17**, 57-61 (1999).
18. Sun, A. *et al.* Potent non-nucleoside inhibitors of the measles virus RNA-dependent RNA polymerase complex. *J Med Chem* **51**, 3731-3741 (2008).
